# Prediction Models for Agonists and Antagonists of Molecular Initiation Events for Toxicity Pathways Using an Improved Deep-Learning-Based Quantitative Structure–Activity Relationship System

**DOI:** 10.3390/ijms221910821

**Published:** 2021-10-06

**Authors:** Yasunari Matsuzaka, Shin Totoki, Kentaro Handa, Tetsuyoshi Shiota, Kota Kurosaki, Yoshihiro Uesawa

**Affiliations:** 1 Department of Medical Molecular Informatics, Meiji Pharmaceutical University, Kiyose, Tokyo 204-8588, Japan; yasunari80808@ims.u-tokyo.ac.jp (Y.M.); d196955@std.my-pharm.ac.jp (K.K.); 2 Center for Gene and Cell Therapy, Division of Molecular and Medical Genetics, The Institute of Medical Science, University of Tokyo, Minato-ku, Tokyo 108-8639, Japan; 3 Fujitsu Limited, Kawasaki-shi, Kanagawa 211-8588, Japan; totoki.shin@fujitsu.com (S.T.); handa.kentaro@fujitsu.com (K.H.); shiota@fujitsu.com (T.S.)

**Keywords:** chemical structure, DeepSnap, deep learning, nuclear receptor, QSAR, Tox21

## Abstract

In silico approaches have been studied intensively to assess the toxicological risk of various chemical compounds as alternatives to traditional in vivo animal tests. Among these approaches, quantitative structure–activity relationship (QSAR) analysis has the advantages that it is able to construct models to predict the biological properties of chemicals based on structural information. Previously, we reported a deep learning (DL) algorithm-based QSAR approach called DeepSnap-DL for high-performance prediction modeling of the agonist and antagonist activity of key molecules in molecular initiating events in toxicological pathways using optimized hyperparameters. In the present study, to achieve high throughput in the DeepSnap-DL system–which consists of the preparation of three-dimensional molecular structures of chemical compounds, the generation of snapshot images from the three-dimensional chemical structures, DL, and statistical calculations—we propose an improved DeepSnap-DL approach. Using this improved system, we constructed 59 prediction models for the agonist and antagonist activity of key molecules in the Tox21 10K library. The results indicate that modeling of the agonist and antagonist activity with high prediction performance and high throughput can be achieved by optimizing suitable parameters in the improved DeepSnap-DL system.

## 1. Introduction

Recently, methods for predicting toxicity risk have focused on deep learning (DL)-based models, due to their high accuracy and the use of large datasets, as alternatives to animal testing. These methods follow the principle of the 3Rs (replacement, reduction, and refinement) for the discovery of molecular initiating events (MIEs) in the adverse outcome pathway (AOP), where MIEs are the first point of chemical–biological interaction within the human body [1,2,3]. The interaction between endocrine-disrupting chemicals and nuclear–receptor family proteins can affect the endocrine system in the AOP [4]. The Toxicology in the 21st Century (Tox21) program is a US federal research collaboration between the US Environmental Protection Agency, the National Toxicology Program, the National Center for Advancing Translational Sciences, and the Food and Drug Administration that aims to develop toxicity assessment methods for commercial chemicals, pesticides, food additives/contaminants, and medical products using quantitative high-throughput screening [5,6,7]. The Tox21 10K library consists of approximately 10,000 (10K) chemicals, including nearly 100 million data points obtained by in vitro quantitative high-throughput screening, indicating the toxicological risk of chemical compounds obtained using an in silico approach [8,9]. Using Tox21 data, the quantitative structure–activity relationship (QSAR) competition organized by Merck—referred to as the Tox21 Data Challenge 2014—demonstrated high-performance prediction modeling using machine learning (ML) [10]. However, ML has limitations if the quality and quantity of the data are insufficient [3,11,12].

Recently, a QSAR approach using DL has demonstrated improvements in terms of time and cost for toxicity prediction using complex nonlinear models of chemical compounds as an in silico approach that can be used as an alternative to animal testing [13,14,15]. In this approach, DL with a specific deep-neural-network architecture that has many layers of hidden neurons with backpropagation and stochastic gradient descent can automatically extract high-dimensional features and heterogeneous chemical data to avoid overfitting without using descriptors [16,17,18,19,20,21,22]. 

As a recent state-of-the-art method, graph convolutional neural networks—which use a graphical representation of molecules as a more natural framework, embedding nodes in a graph into Euclidean space—have been applied to chemical-network predictions using two-dimensional (2D) molecular graphs without chemical descriptors [16,23,24,25]. Unlike conventional methods that use descriptors, an elementary portrait of each molecule using a graphical representation with each node and edge of the graph representing an atom and bond, respectively, can avoid overfitting in ML algorithms with an excessive number of descriptors [26,27,28]. Although this approach has higher performance than conventional methods, there are several problems. One problem is that the representation of all nodes converges to the same value, along with repetitions of the graph convolution operation when the number of layers in the graph convolutional neural network is increased, resulting in reduced performance [29,30,31]. Another problem is that label information cannot be propagated to the entire graph when there is limited label information, resulting in low performance [32,33,34].

A deep feedforward neural network, which calculates the weights and biases of many hidden layers and uses a bounded number of hidden neurons with precalculated molecular descriptors as inputs, has become popular because it can approximate any continuous function on a compact subset of R^n^; this is called the universal approximation theorem [3,20,35]. Although the learning speed of this neural network is higher due to the calculation of a gradient for a randomly selected training dataset and the sequential update of the parameters, it has the drawback of imprecision: a small neural network can lead to underfitting, while a large neural network can lead to overfitting [20,36].

A DL-based QSAR approach—which can capture detailed information from three-dimensional (3D) molecular structures by including their rotation angles to establish a systematic method of inputting molecular structures—has also been reported [37]. In a previous study, we reported 35 prediction models for agonists and antagonists of nuclear receptors as MIEs for chemical compounds derived from the Tox21 10K library using an approach called DeepSnap-DL. Of these prediction models, three models exhibited higher performance than the best-performing models in the Tox21 Data Challenge 2014 [38]. However, DeepSnap-DL is a complex system that consists of four steps: the preparation of a 3D molecular structure from one-dimensional chemical information, generation of a snapshot image of the 3D chemical structure, DL, and statistical calculations. 

In the current study, we present an improved DeepSnap-DL approach that is a one-step system that sequentially executes DeepSnap, which is a process that depicts 3D ball-and stick models with different colors representing different atoms and finally captures snapshots automatically in selected angle increments on the *x*, *y*, and *z* axes and saves 256 × 256-pixel resolution PNG files with RGB colors into three types of datasets: the training (train), validation (valid), and test datasets. The DL is improved using TensorFlow, a free and open-source software library for ML. It is particularly useful for the training of and inference from deep neural networks that can run on multiple central processing units and graphics processing units (GPUs). It employs optional Compute Unified Device Architecture and a higher-level programming model to improve programming productivity on various hardware accelerators. It also includes SYCL, which is a royalty-free, higher-level programming model to improve programming productivity on various hardware accelerators and extensions for general-purpose computing on GPUs and employs Keras, an open-source software library that provides a Python interface for artificial neural networks and acts as an interface for the TensorFlow library as well as for high-throughput statistical calculations. We constructed a total of 59 prediction models using the improved DeepSnap-DL system and the Tox21 10K library, and the results indicate that the improved DeepSnap-DL approach has higher throughput than the previous DeepSnap-DL approach. Furthermore, the models constructed using the improved DeepSnap-DL system can achieve high prediction performance by optimizing the parameter choices.

## 2. Results

### 2.1. Prediction Models for 59 MIEs

We have previously reported a DL-based QSAR approach, called the DeepSnap-DL- system, which uses the Deep Learning GPU Training System (DIGITS) [37,38,39,40,41]. In the current study, we present an improved DeepSnap-DL system that uses TensorFlow and Keras. In this system, the flow of image generation and learning is regarded as one unit that can be executed sequentially and automatically to obtain high throughput and high performance (Figure S1).

To construct prediction models of MIEs for in vivo toxicity responses that affect health outcomes in humans, we downloaded information about the chemical structures and agonist and antagonist activity for a total of 59 MIEs from the Tox21 10K library. The mean number of chemicals in the 59 MIEs was 9699 ± 702, and the highest and lowest numbers of chemicals were, respectively, 12,581 (for the peroxisome proliferator-activated receptor γ antagonist: PPARg_ant, AID: 743199, where AID is the agonist/antagonist identification number, and the antioxidant response element signaling pathway agonist: ARE_ago, AID: 743219) and 8434 (the caspase-2 agonist: Casp2_ago, AID: 1347037) (Figure S2a). Furthermore, we divided the data for these chemical compounds into two groups based on their activity scores: active chemicals had an activity score ≥ 40, while inactive chemicals had an activity score < 40. The mean percentage of active chemicals among all chemicals was 4.79% ± 3.94%, and the highest and lowest percentages of active chemicals were 21.6% (for the pregnane X receptor agonist: PXR_ago, AID: 1347033) and 0.08% (the transforming growth factor–β agonist: TGFb_ago, AID: 1347035), respectively (Figure S2b). The data were divided into training, validation, test, and foldout datasets, with the first three datasets used for training and fine-tuning the prediction models. The final evaluation of the constructed models was performed using the foldout dataset.

The mean number of chemicals in the training, validation, and test datasets for the 59 MIEs was 7046 ± 608, and the highest and lowest numbers of chemicals were 9834 (AID: 743199 and AID: 743219) and 6388 (the peroxisome proliferator-activated receptor δ agonist: PPARd_ago, AID: 743227), respectively (Figure S3a). The mean percentage of active chemicals in the training, validation, and test datasets was 4.77% ± 3.93%, and the highest and lowest percentages of active chemicals were 21.6% (AID: 1347033) and 0.08% (AID: 1347035), respectively (Figure S3b). In addition, the mean number of chemicals in the foldout dataset of the 59 MIEs was 2654 ± 469, and the highest and lowest numbers of chemicals were 3101 (the ATPase family AAA-domain-containing protein 5 agonist: ATAD5_ago, AID: 720516; the glucocorticoid receptor agonist: GR_ago, AID: 720719; the glucocorticoid receptor antagonist: GR_ant, AID: 720725; the androgen receptor agonist: ARlbd_ago, AID: 743053; the thyroid hormone receptor beta isoform 2 antagonist: TR_ant, AID: 743067; the estrogen nuclear receptor alpha *agonist:* *ERlbd_ago*, AID: 743077; the estrogen nuclear receptor alpha antagonist: ERlbd_ant, AID: 743078; and the estrogen nuclear receptor alpha antagonist: ERfull_ant, AID: 743091) and 843 (AID: 1347037), respectively (Figure S4a). The mean percentage of active chemicals in the foldout dataset was 4.85% ± 4.16%, and the highest and lowest percentages of active chemicals were 21.7% (the pregnane X receptor agonist: PXR_ago, AID: 1347033) and 0.10% (AID: 1347035), respectively (Figure S4b). These results indicate that the datasets were highly imbalanced.

Next, using structural information for these chemicals derived from the simplified molecular-input line-entry system (SMILES) format, the SMILES_TO_SDF (Structure Data File) program, which performs conformational import from the SMILES format, produced 3D chemical conformation structures. Molecular images were then generated as snapshots of the 3D structure using the improved DeepSnap-DL method at six angles along the *x*, *y*, and *z* axes. This produced the following different numbers of images: (195°, 195°, 195°), (8 images); (185°, 185°, 185°), (8 images); (176°, 176°, 176°), (27 images); (165°, 165°, 165°), (27 images); (155°, 155°, 155°), (27 images); and (145°, 145°, 145°), (27 images). Using these molecular images as input data for DL, we constructed a total of 59 prediction models for agonists or antagonists of the MIEs and fine-tuned them using the modified DeepSnap-DL system and the training, validation, and test datasets. Finally, we selected the models with the highest performance out of the six angles and evaluated them using the foldout dataset. Among the 59 prediction models, one model (the nuclear factor-kappa B agonist: NFkB_ago, AID: 1159518) did not demonstrate predictive ability (ROC_AUC_valid = 0.5, ROC_AUC_test = 0.5, ROC_AUC_foldout = 0.5, where ROC_AUC_valid, ROC_AUC_test, and ROC_AUC_foldout refer to the area under the corresponding receiver operating characteristic curve in the validation, test, and foldout datasets, respectively). The dataset used in the NFkB_ago prediction model was highly imbalanced. This suggests that the performance of the prediction model may depend on the balance of the input data, as previously reported [42,43]. 

**Figure 1:**
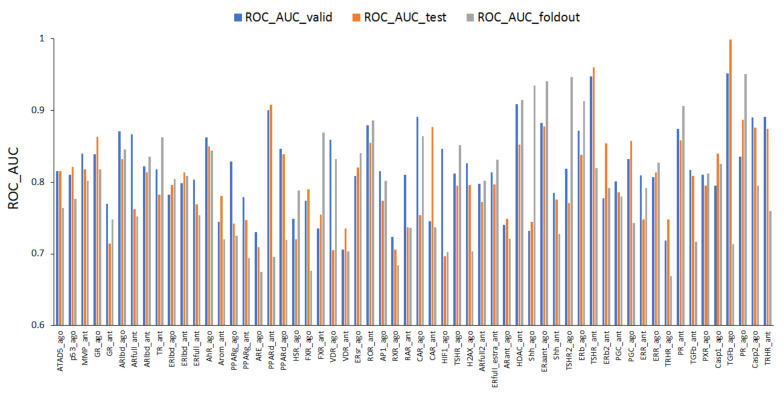
Area under the receiver operating characteristic curve (ROC_AUC) for 59 key molecules in MIEs on the validation, test, and foldout datasets. The model exhibiting the highest prediction performance was selected out of six angles in DeepSnap-DL.

**Figure 2:**
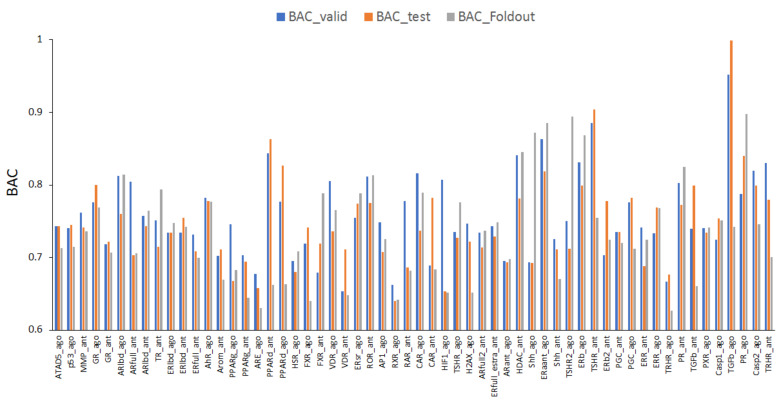
Balanced accuracy (BAC) of 59 key molecules in MIEs on the validation, test, and foldout datasets. The model exhibiting the highest prediction performance was selected out of six angles in DeepSnap-DL.

The mean values of ROC_AUC_valid, ROC_AUC_test, and ROC_AUC_foldout were 0.818 ± 0.056, 0.803 ± 0.063, and 0.792 ± 0.076, respectively (Figure 1). In addition, as illustrated in Figure 1, the highest prediction performance was observed for the transforming growth factor-β (TGFb) agonist (TGFb_ago, AID: 1347035), with ROC_AUC_valid = 0.952 and ROC_AUC_test = 0.999, and for the progesterone receptor agonist (PR_ago, AID: 1347036), with ROC_AUC_foldout = 0.951. The lowest prediction performance was observed for the vitamin D receptor (VDR) antagonist (VDR_ant, AID: 743242), with ROC_AUC_valid = 0.706, the hypoxia-inducible factor-1 agonist (HIF1_ago, AID: 1224894), with ROC_AUC_test = 0.694, and the thyrotropin-releasing hormone receptor agonist (TRHR_ago, AID: 1347030), with ROC_AUC_foldout = 0.669 (Figure 1). Furthermore, with respect to the balanced accuracy (BAC), the mean values of BAC_valid, BAC_test, and BAC_foldout where BAC_valid, BAC_test, and BAC_foldout refer to the area-balanced accuracy in the validation, test, and foldout datasets, respectively, were 0.757 ± 0.058, 0.747 ± 0.062, and 0.736 ± 0.069, respectively (Figure 2). In addition, as illustrated in Figure 2, the highest prediction performance was observed for the TGFb agonist (TGFb_ago, AID: 1347035), with BAC_valid = 0.952 and BAC_test = 0.999, and for the PR agonist (PR_ago, AID: 1347036), with BAC_foldout = 0.898. The lowest prediction performance was observed for the VDR antagonist (VDR_ant, AID: 743242), with BAC_valid = 0.653, the retinoid X receptor (RXR) agonist (RXR_ago, AID: 1159531), with BAC_test = 0.640, and the TRHR agonist (TRHR_ago, AID: 1347030), with BAC_foldout = 0.627 (Figure 2). 

The mean values of specificity_valid, specificity_test, and specificity_foldout, where specificity_valid, specificity_test, and specificity_foldout refer to the specificity in the validation, test, and foldout datasets, respectively, were 0.755 ± 0.100, 0.754 ± 0.111, and 0.756 ± 0.107, respectively (Figure S5). In addition, as illustrated in Figure S5, the highest prediction performance was observed for the HIF1 agonist (HIF1_ago, AID: 1224894), with specificity_valid = 0.980, and for the TGFb agonist (TGFb_ago, AID: 1347035), with specificity_test = 0.997 and specificity_foldout = 0.980. The lowest prediction performance was observed for the sonic hedgehog antagonist (Shh_ago, AID: 1259390), with specificity_valid = 0.490, the VDR antagonist (VDR_ant, AID: 743242), with specificity_test = 0.450, and the androgen-receptor agonist (ARant_ago, AID: 1259387), with specificity_foldout = 0.500 (Figure S5). The mean values of recall_valid, recall_test, and recall_foldout—where recall_valid, recall_test, and recall_foldout, refer to the recall in the validation, test, and foldout datasets, respectively—were 0.760 ± 0.086, 0.741 ± 0.113, and 0.709 ± 0.132, respectively (Figure S6). In addition, as illustrated in Figure S6, the highest prediction performance was observed for the TGFb agonist (TGFb_ago, AID: 1347035), with recall_valid = 1.00 and recall_test = 1.00, and for the thyroid-stimulating hormone receptor 2 agonist (TSHR2_ago, AID: 1259393), with recall_foldout = 0.930. The lowest prediction performance was observed for the VDR antagonist (VDR_ant, AID: 743242), with recall_valid = 0.610, the RXR agonist (RXR_ago, AID: 1159531), with recall_test = 0.470, and the farnesoid X receptor agonist (FXR_ago, AID: 743239), with recall_foldout = 0.040 (Figure S6). 

In a previous study, we reported the construction of prediction models for 35 agonist and antagonist allosteric modulators of nuclear receptors for chemicals using the DeepSnap-DL system with DIGITS [38]. That study reported that the mean ROC_AUC and BAC were 0.884 ± 0.017 and 0.8471 ± 0.017, respectively [38]. The results of the present study indicate that the prediction performance of DeepSnap-DL with TensorFlow and Keras is lower than that of DeepSnap-DL with DIGITS. However, DeepSnap-DL with TensorFlow and Keras has the potential for improvement through detailed examinations using different angles.

### 2.2. Angles and Data Split in DeepSnap-DL

**Figure 3:**
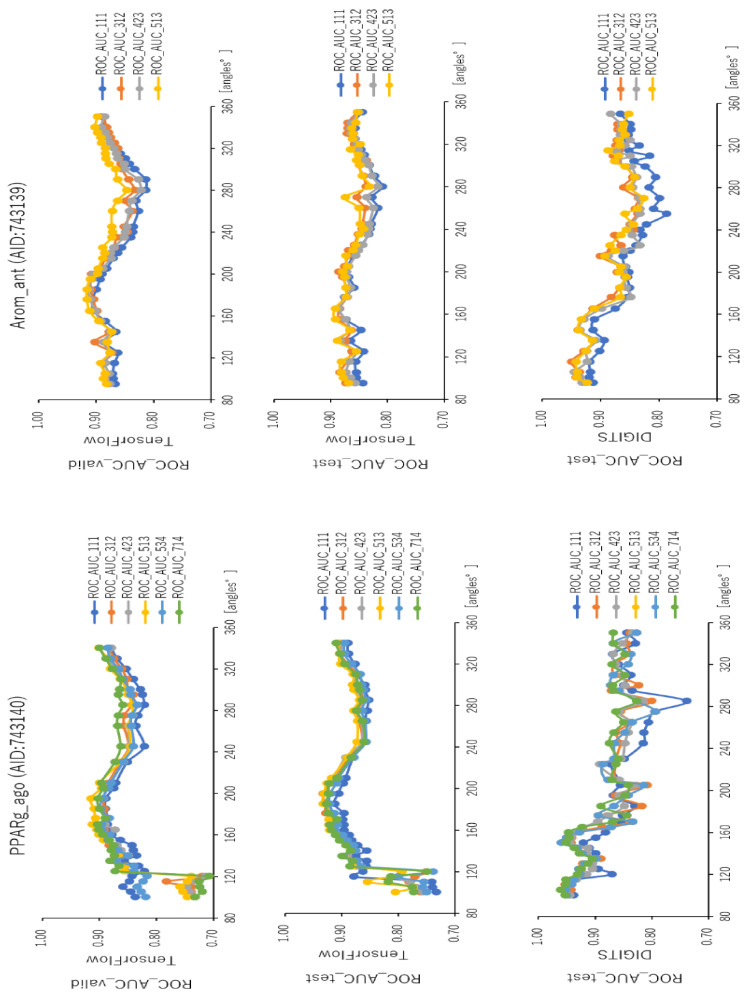
ROC_AUC for PPARg_ago (AID:743140) and Arom_ant (AID:743139) on the validation and test datasets using TensorFlow and Keras in the test dataset using DIGITS with 31 angles in DeepSnap-DL. The different curves represent different ratios of splits among the dataset, training:validation:test =1:1:1, 3:1:2, 4:2:3, 5:1:3, 5:3:4, and 7:1:4, respectively. Three-fold experiments were performed.

**Figure 4:**
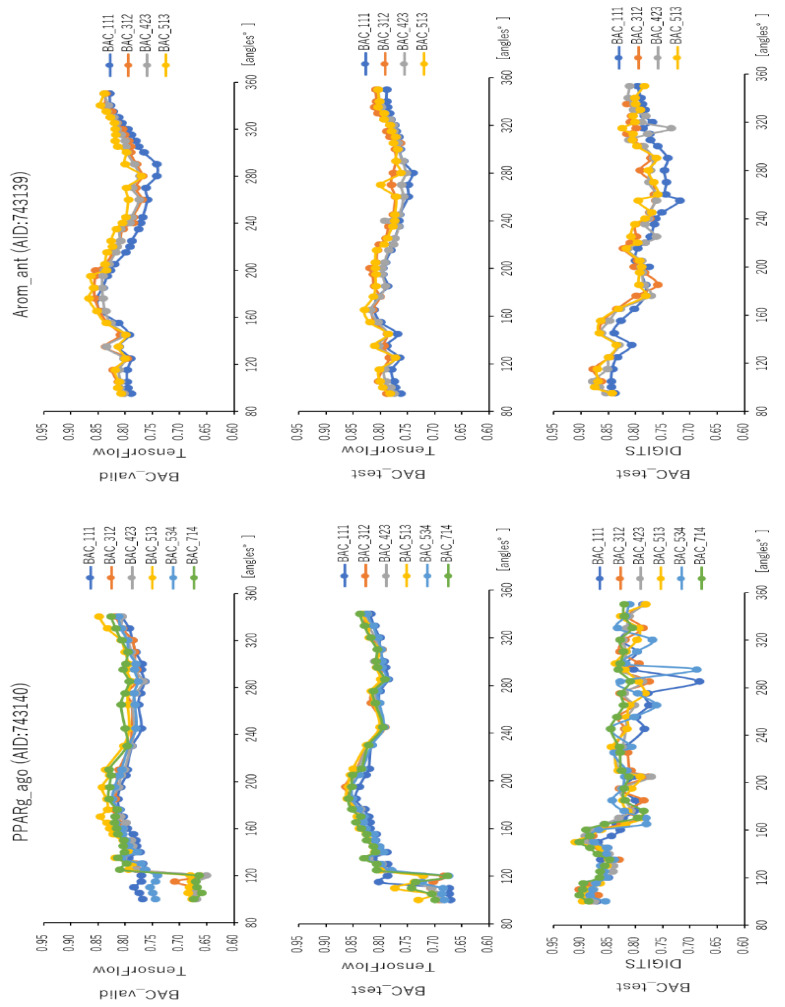
BAC for PPARg_ago (AID:743140) and Arom_ant (AID:743139) on the validation and test datasets using TensorFlow and Keras on the test dataset using DIGITS with 31 angles in DeepSnap-DL. The different curves represent different ratios of the splits among the datasets, training:validation:test =1:1:1, 3:1:2, 4:2:3, 5:1:3, 5:3:4, and 7:1:4, respectively. Three-fold experiments were performed.

**Figure 5:**
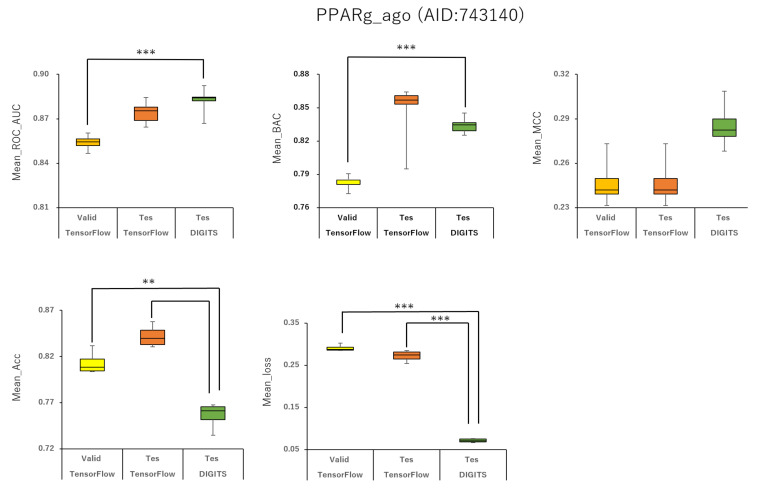
Means ROC_AUC, means BAC, means MCC, means Acc, and means loss of PPARg_ago (AID:743140) in validation and test datasets in TensorFlow and test dataset in DIGITS. **p < 0.01, ***p < 0.001 for *t*-test.

**Figure 6:**
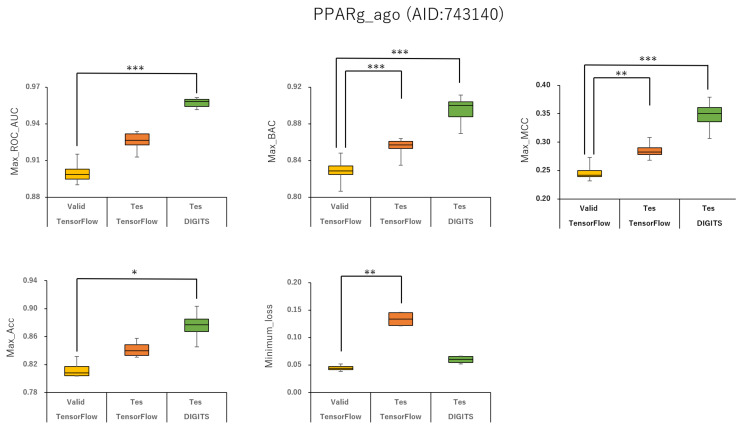
Max ROC_AUC, max BAC, max MCC, max Acc, and minimum loss of PPARg_ago (AID:743140) in validation and test datasets in TensorFlow and test dataset in DIGITS. *p < 0.05, **p < 0.01, ***p < 0.001 for *t*-test.

**Figure 7:**
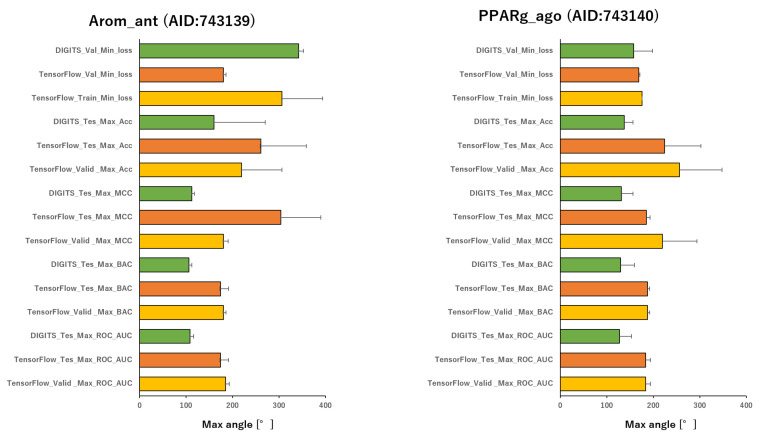
Means angles of max_ROC_AUC, max_BAC, max_MCC, max_Acc, min_loss of Arom_ant (AID:743139) and PPARg_ago (AID;743140).

To analyze the influence of different angles on the snapshot generation of DeepSnap and of different splits among the training, validation, and test datasets of the peroxisome proliferator-activated receptor γ(PPARγ) agonist (PPARg_ago, AID:743140), we used 31 different angles from (100°, 100°, 100°) (64 images) to (340°, 340°, 340°) (8 images), and six types of data-split ratios (training:validation:test = 1:1:1, 3:1:2, 4:2:3, 5:1:3, 5:3:4, 7:1:4) in the improved DeepSnap-DL using TensorFlow and Keras. The mean ROC_AUC, BAC, Matthews correlation coefficient (MCC), and accuracy (Acc) values in the validation and test datasets for the 31 angles and six data-split ratios were 0.854 ± 0.005 (ROC_AUC_valid), 0.874 ± 0.007 (ROC_AUC_test), 0.783 ± 0.006 (BAC_valid), 0.848 ± 0.026 (BAC_test), 0.237 ± 0.026 (MCC_valid), 0.273 ± 0.035 (MCC_test), 0.799 ± 0.035 (Acc_valid), and 0.827 ± 0.043 (Acc_test), where MCC_valid, MCC_test, Acc_valid, and Acc_test refer to the MCC and the accuracy in the validation and test datasets, respectively. The mean loss values on the training and validation datasets were 0.287 ± 0.110 (loss_train) and 0.270 ± 0.076 (loss_valid), where loss_train and loss_valid refer to the loss in the training and validation datasets (Figure 3, Figure 4, Figure 5, Figure 6 and Figure 7; Figures S7–S10, left; Table S1). The highest prediction performance values on the validation and test datasets for the 31 angles and six data-split ratios were 0.915 at 195° (ROC_AUC_valid), 0.934 at 195° (ROC_AUC_test), 0.848 at 185° (BAC_valid), 0.864 at 195° (BAC_test), 0.273 at 176° (MCC_valid), 0.309 at 176° (MCC_test), 0.832 at 170° (Acc_valid), and 0.858 at 176° (Acc_test). The lowest prediction performance values on the training and validation datasets were 0.038 at 176°(loss_train) and 0.122 at 165°and 170° (loss_valid) (Figure 3, Figure 4, Figure 5, Figure 6 and Figure 7; Figures S7–S10, left; Table S1). 

In addition, we used 34 angles from (100°, 100°, 100°) (64 images) to (350°, 350°, 350°) (8 images) and six data-split ratios (training:validation:test = 1:1:1, 3:1:2, 4:2:3, 5:1:3, 5:3:4, and 7:1:4) in DeepSnap-DL with DIGITS [38,39,40,41]. The mean ROC_AUC, BAC, MCC, and Acc values on the test dataset for the 34 angles and six data-split ratios were 0.882 ± 0.008 (ROC_AUC_test), 0.834 ± 0.007 (BAC_test), 0.245 ± 0.009 (MCC_test), and 0.751 ± 0.016 (Acc_test). The mean loss value on the validation dataset was 0.073 ± 0.006 (loss_valid) (Figure 3, Figure 4, Figure 5, Figure 6 and Figure 7; Figures S7–S10, left; Table S1). The highest prediction performance values on the test dataset for the 34 angles and six data-split ratios were 0.962 at 105° (ROC_AUC_test), 0.912 at 150° (BAC_test), 0.379 at 115° (MCC_test), and 0.903 at 115° (Acc_test). The lowest prediction performance value on the validation dataset was 0.052 at 176° (loss_valid) (Figure 3, Figure 4, Figure 5, Figure 6 and Figure 7; Figures S7–S10, left; Table S1).

**Figure 8:**
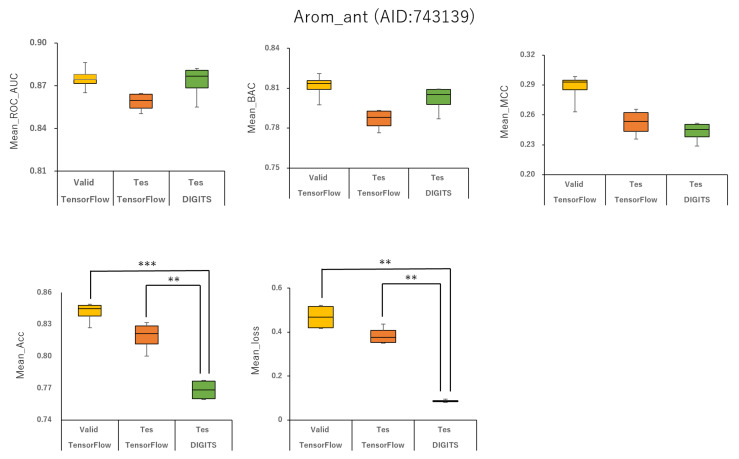
Means ROC_AUC, means BAC, means MCC, means Acc, and means loss of Arom_ant (AID:743139) in validation and test datasets in TensorFlow and test dataset in DIGITS. **p < 0.01, ***p < 0.001 for *t*-test.

**Figure 9:**
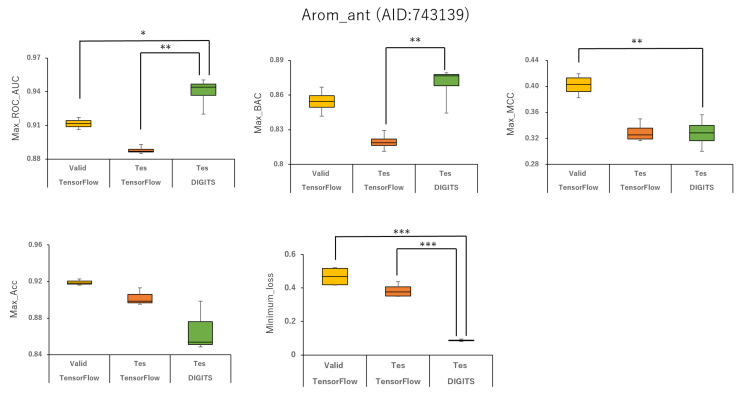
Max ROC_AUC, max BAC, max MCC, max Acc, and minimum loss of Arom_ant (AID:743139) in validation and test datasets in TensorFlow and test dataset in DIGITS. **p < 0.01, ***p < 0.001 for *t*-test.

Furthermore, using the aromatase antagonist (Arom_ant, AID:743139), we built prediction models for 34 angles from (95°, 95°, 95°) (64 images) to (350°, 350°, 350°) (8 images) and four data-split ratios (training:validation:test = 1:1:1, 3:1:2, 4:2:3, and 5:1:3) in DeepSnap-DL using TensorFlow and Keras. The mean values of ROC_AUC, BAC, MCC, and Acc on the validation and test datasets for the 34 angles and four data-split ratios were 0.875 ± 0.009 (ROC_AUC_valid), 0.859 ± 0.007 (ROC_AUC_test), 0.811 ± 0.010 (BAC_valid), 0.786 ± 0.008 (BAC_test), 0.287 ± 0.016 (MCC_valid), 0.252 ± 0.014 (MCC_test), 0.841 ± 0.010 (Acc_valid), and 0.819 ± 0.014 (Acc_test). The mean loss values on the training and validation datasets were 0.469 ± 0.125 (loss_train) and 0.385 ± 0.080 (loss_valid) (Figure 3, Figure 4, Figure 7, Figure 8 and Figure 9; Figures S7–S10, right; Table S2). The highest prediction performance values on the validation and test datasets for the 34 angles and four data-split ratios were 0.917 at 185° (ROC_AUC_valid), 0.893 at 165° (ROC_AUC_test), 0.867 at 176° (BAC_valid), 0.830 at 165° (BAC_test), 0.420 at 176° (MCC_valid), 0.350 at 350° (MCC_test), 0.929 at 176° (Acc_valid), and 0.913 at 176° (Acc_test). The lowest prediction-performance values on the training and validation datasets were 0.022 at 350° (loss_train) and 0.102 at 185° (loss_valid) (Figure 3, Figure 4, Figure 7, Figure 8 and Figure 9; Figures S7–S10, right; Table S2).

In addition, we used 34 angles from (95°, 95°, 95°) (64 images) to (350°, 350°, 350°) (8 images) and four data-split ratios (training:validation:test = 1:1:1, 3:1:2, 4:2:3, and 5:1:3) in DeepSnap-DL using DIGITS. The mean values of ROC_AUC, BAC, MCC, and Acc on the test dataset for the 34 angles and four data-split ratios were 0.873 ± 0.012 (ROC_AUC_test), 0.802 ± 0.010 (BAC_test), 0.243 ± 0.010 (MCC_test), and 0.768 ± 0.010 (Acc_test). The mean loss value on the validation dataset was 0.087 ± 0.007 (loss_valid) (Figure 3 and Figure 4; Figures S7–S10, right; Table S2). The highest prediction performance values on the test dataset for the 34 angles and four data-split ratios were 0.950 at 115° (ROC_AUC_test), 0.879 at 105° (BAC_test), 0.356 at 105° (MCC_test), and 0.899 at 105° (Acc_test). The lowest prediction performance value on the validation dataset was 0.069 at 340°and 350° (loss_valid) (Figure 3, Figure 4, Figure 7, Figure 8 and Figure 9; Figure S7–S10, right; Table S2).

### 2.3. Learning Rate and Batch Size in DeepSnap-DL

**Figure 10:**
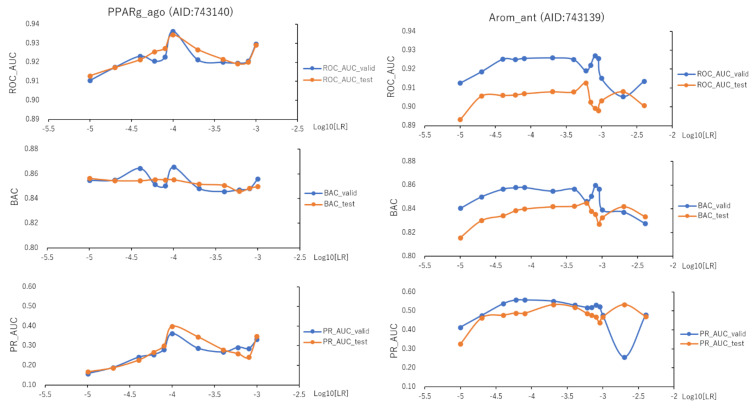
ROC_AUC, BAC, and PR_AUC for PPARg_ago (AID:743140) and Arom_ant (AID:743139) on the validation and test datasets using TensorFlow with different learning rates (LRs). Three-fold experiments were performed.

To assess the effect of the hyperparameters in the DeepSnap-DL system on the prediction performance, we optimized 11 learning rates (LRs) from 0.001 to 0.00001 of PPARg_ago using the validation and test datasets (Figure 10, left; Figure S11, left; Table S3). The mean values of ROC_AUC, BAC, PR_AUC (the area under the precision–recall curve), MCC, and Acc on the validation and test datasets for the 11 LRs were 0.916 ± 0.022 (ROC_AUC_valid), 0.916 ± 0.025 (ROC_AUC_test), 0.848 ± 0.018 (BAC_valid), 0.847 ± 0.020 (BAC_test), 0.253 ± 0.077 (PR_AUC_valid), 0.258 ± 0.089 (PR_AUC_test), 0.864 ± 0.024 (F_valid, which is the F-measure (F) value in the validation dataset), 0.868 ± 0.023 (F_tes—which is the F value in the test dataset), 0.791 ± 0.036 (Acc_valid), 0.799 ± 0.036 (Acc_test), 0.259 ± 0.097 (loss_train), 0.239 ± 0.107 (loss_valid), 0.239 ± 0.102 (loss_test), 0.255 ± 0.023 (MCC_valid), and 0.258 ± 0.025 (MCC_test) (Figure 10, left; Figure S11, left; Table S3). The highest prediction performance values on the validation and test datasets for the 11 LRs were 0.936 at LR = 0.0001 (ROC_AUC_valid), 0.934 at LR = 0.0001 (ROC_AUC_test), 0.865 at LR = 0.0001 (BAC_valid), 0.856 at LR = 0.00001 (BAC_test), 0.362 at LR = 0.0001 (PR_AUC_valid), 0.399 at LR = 0.0001 (PR_AUC_test), 0.888 at LR = 0.0001 (F_valid), 0.903 at LR = 0.0001 (F_test), 0.830 at LR = 0.0001 (Acc_valid), 0.854 at LR = 0.0001 (Acc_test), 0.286 at LR = 0.0001 (MCC_valid), and 0.293 at LR = 0.0001 (MCC_test) (Figure 10, left; Figure S11, left; Table S3). The lowest prediction performance values on the training, validation, and test datasets for the 11 LRs were 0.157 at LR = 0.0001 (loss_train), 0.131 at LR = 0.0001 (loss_valid), and 0.147 at LR = 0.0002 (loss_test) (Figure S11, left; Table S3).

In addition, we fine-tuned 14 LRs from 0.004 to 0.00001 of Arom_ant using the validation and test datasets (Figure 10, right; Figure S11, right; Table S4). The mean values of ROC_AUC, BAC, PR_AUC, MCC, and Acc on the valid and test datasets for the 14 LRs were 0.920 ± 0.007 (ROC_AUC_valid), 0.904 ± 0.005 (ROC_AUC_test), 0.849 ± 0.010 (BAC_valid), 0.835 ± 0.008 (BAC_test), 0.495 ± 0.079 (PR_AUC_valid), 0.474 ± 0.050 (PR_AUC_test), 0.902 ± 0.017 (F_valid), 0.902 ± 0.005 (F_test), 0.858 ± 0.027 (Acc_valid), 0.858 ± 0.008 (Acc_test), 0.211 ± 0.048 (loss_train), 0.189 ± 0.043 (loss_valid), 0.163 ± 0.043 (loss_test), 0.329 ± 0.030 (MCC_valid), and 0.412 ± 0.011 (MCC_test) (Figure 10, right; Figure S11, right; Table S4). The highest prediction performance values on the validation and test datasets for the 14 LRs were 0.927 at LR = 0.0008 (ROC_AUC_valid), 0.913 at LR = 0.0006 (ROC_AUC_test), 0.860 at LR = 0.0008 (BAC_valid), 0.845 at LR = 0.0006 (BAC_test), 0.557 at LR = 0.00008 (PR_AUC_valid), 0.533 at LR = 0.0002 (PR_AUC_test), 0.925 at LR = 0.00008 (F_valid), 0.845 at LR = 0.0006 (F_test), 0.895 at LR = 0.00008 (Acc_valid), 0.872 at LR = 0.0004 (Acc_test), 0.381 at LR = 0.0008 (MCC_valid), and 0.331 at LR = 0.0004 (MCC_test) (Figure 10, right; Figure S11, right; Table S4). The lowest prediction performance values on the training, validation, and test datasets for the 14 LRs were 0.138 at LR = 0.00008 (loss_train), 0.125 at LR = 0.00006 (loss_valid), and 0.113 at LR = 0.0002 (loss_test) (Figure S11, right; Table S4).

**Figure 11:**
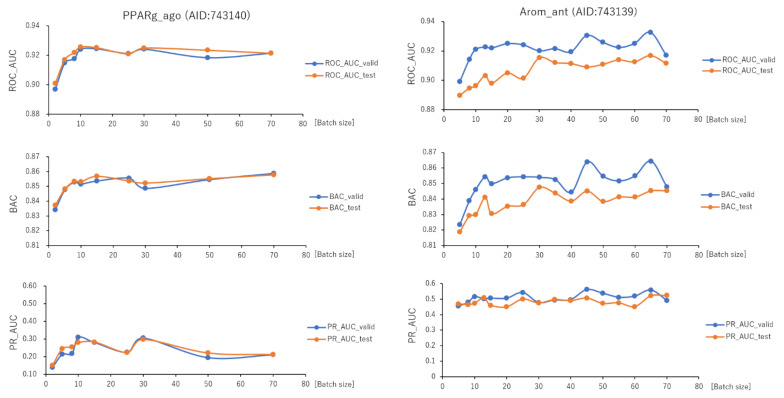
ROC_AUC, BAC, and PR_AUC for PPARg_ago (AID:743140) and Arom_ant (AID:743139) using TensorFlow with different batch sizes. Three-fold experiments were performed.

To investigate the effect of the batch size (BS) in the improved DeepSnap-DL system on prediction performance, we optimized a total of nine BSs from PPARg_ago, from 2 to 70, using the validation and test datasets (Figure 11, left; Figure S12, left; Table S5). The mean values of ROC_AUC, BAC, PR_AUC, F, Acc, loss, and MCC on the validation and test datasets for the nine BSs were 0.918 ± 0.009 (ROC_AUC_valid), 0.920 ± 0.008 (ROC_AUC_test), 0.851 ± 0.007 (BAC_valid), 0.852 ± 0.006 (BAC_test), 0.233 ± 0.055 (PR_AUC_valid), 0.241 ± 0.045 (PR_AUC_test), 0.868 ± 0.012 (F_valid), 0.870 ± 0.009 (F_test), 0.798 ± 0.018 (Acc_valid), 0.801 ± 0.014 (Acc_test), 0.276 ± 0.084 (loss_train), 0.238 ± 0.081 (loss_valid), 0.213 ± 0.052 (loss_test), 0.259 ± 0.011 (MCC_valid), and 0.260 ± 0.010 (MCC_test) (Figure 11, left; Figure S12, left; Table S5). The highest prediction performance values on the validation and test datasets for the nine BSs were 0.924 at BS = 15 (ROC_AUC_valid), 0.926 at BS = 10 (ROC_AUC_test), 0.859 at BS = 70 (BAC_valid), 0.858 at BS = 70 (BAC_test), 0.309 at BS = 10 (PR_AUC_valid), 0.298 at BS = 30 (PR_AUC_test), 0.884 at BS = 30 (F_valid), 0.882 at BS = 10 (F_test), 0.823 at BS = 30 (Acc_valid), 0.820 at BS = 10 (Acc_test), 0.269 at BS = 30 (MCC_valid), and 0.270 at BS = 10 (MCC_test) (Figure 11, left; Figure S12, left; Table S5). The lowest prediction performance values on the training, validation, and test datasets for the nine BSs were 0.160 at BS = 30 (loss_train), 0.143 at BS = 2 (loss_valid), and 0.140 at BS = 30 (loss_test) (Figure S12, left; Table S5).

In addition, we fine-tuned a total of 16 BSs from 5 to 70 of Arom_ant using the validation and test datasets (Figure 11, right; Figure S12, right; Table S6). The mean values of ROC_AUC, BAC, PR_AUC, F, Acc, loss, and MCC on the validation and test datasets for the 16 BSs were 0.921 ± 0.007 (ROC_AUC_valid), 0.906 ± 0.008 (ROC_AUC_test), 0.851 ± 0.010 (BAC_valid), 0.838 ± 0.008 (BAC_test), 0.509 ± 0.029 (PR_AUC_valid), 0.484 ± 0.024 (PR_AUC_test), 0.903 ± 0.010 (F_valid), 0.894 ± 0.005 (F_test), 0.860 ± 0.016 (Acc_valid), 0.853 ± 0.008 (Acc_test), 0.198 ± 0.043 (loss_train), 0.193 ± 0.037 (loss_valid), 0.208 ± 0.037 (loss_test), 0.329 ± 0.019 (MCC_valid), and 0.310 ± 0.009 (MCC_test) (Figure 11, right; Figure S12, right; Table S6). The highest prediction performance values on the validation and test datasets for the 16 BSs were 0.933 at BS = 65 (ROC_AUC_valid), 0.917 at BS = 65 (ROC_AUC_test), 0.864 at BS = 65 (BAC_valid), 0.848 at BS = 30 (BAC_test), 0.561 at BS = 45 (PR_AUC_valid), 0.524 at BS = 70 (PR_AUC_test), 0.919 at BS = 13 (F_valid), 0.912 at BS = 45 (F_test), 0.886 at BS = 13 (Acc_valid), 0.874 at BS = 45 (Acc_test), 0.356 at BS = 13 (MCC_valid), and 0.335 at BS = 45 (MCC_test) (Figure 11, right; Figure S12, right; Table S6). The lowest prediction performance values on the training, validation, and test datasets for the 16 BSs were 0.135 at BS = 60 (loss_train), 0.140 at BS = 45 (loss_valid), and 0.148 at BS = 8 (loss_test) (Figure S12, right; Table S6).

### 2.4. Background Colors in Images Produced by DeepSnap-DL

**Figure 12:**
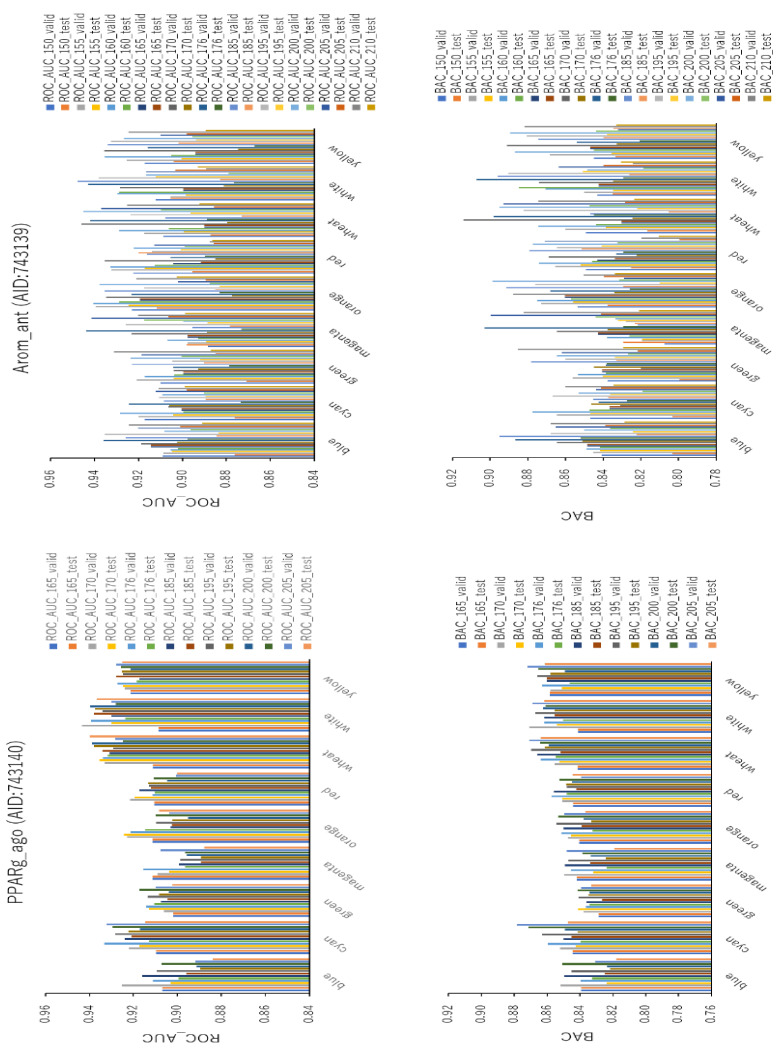
ROC_AUC and BAC for PPARg_ago (AID:743140) and Arom_ant (AID:743139) using TensorFlow with different background colors. Three-fold experiments were performed.

To examine the effect on prediction performance of the background colors of the snapshot images produced by the improved DeepSnap-DL system, we used a total of nine colors (i.e., blue, cyan, green, magenta, orange, red, wheat, white, and yellow) as different background colors, with seven angles (i.e., 165°, 170°, 176°, 185°, 195°, 200°, and 205°) in the validation and test datasets for PPARg_ago (Figure 12, left; Figures S13–S17; Table S7). The mean values of ROC_AUC, BAC, PR_AUC, F, Acc, loss, and MCC on the validation and test datasets for the nine colors with seven angles were 0.917 ± 0.013 (ROC_AUC_valid), 0.914 ± 0.014 (ROC_AUC_test), 0.851 ± 0.012 (BAC_valid), 0.844 ± 0.013 (BAC_test), 0.247 ± 0.081 (PR_AUC_valid), 0.272 ± 0.098 (PR_AUC_test), 0.864 ± 0.021 (F_valid), 0.865 ± 0.024 (F_test), 0.793 ± 0.032 (Acc_valid), 0.794 ± 0.037 (Acc_test), 0.177 ± 0.082 (loss_train), 0.168 ± 0.083 (loss_valid), 0.169 ± 0.057 (loss_test), 0.261 ± 0.021 (MCC_valid), and 0.255 ± 0.023 (MCC_test) (Figure 12, left; Figures S13–S17; Table S7). The highest prediction performance values among the nine colors on the validation and test datasets were 0.932 ± 0.012 for white (ROC_AUC_valid), 0.931 ± 0.010 for wheat (ROC_AUC_test), 0.862 ± 0.010 for white (BAC_valid), 0.857 ± 0.007 for yellow (BAC_test), 0.347 ± 0.091 for white (PR_AUC_valid), 0.384 ± 0.117 for wheat (PR_AUC_test), 0.877 ± 0.023 for wheat (F_valid), 0.891 ± 0.020 for wheat (F_test), 0.813 ± 0.036 for wheat (Acc_valid), 0.836 ± 0.030 for wheat (Acc_test), 0.278 ± 0.024 for white (MCC_valid), and 0.284 ± 0.018 for wheat (MCC_test) (Figure 12, left; Figures S13–S17; Table S7). The lowest prediction performance values on the training, validation, and test datasets for the nine colors were 0.113 ± 0.056 for white (loss_train), 0.111 ± 0.056 for white (loss_valid), and 0.122 ± 0.023 for white (loss_test) (Figure S17 Table S7).

We also used a different nine colors (i.e., blue, cyan, green, magenta, orange, red, wheat, white, and yellow) as background colors with the seven angles (i.e., 150°, 155°, 160°, 165°, 170°, 176°, 185°, 195°, 200°, 205°, and 210°) in the validation and test datasets for Arom_ant (Figure 12, right; Figures S18–S22; Table S8). The mean values of ROC_AUC, BAC, PR_AUC, F, Acc, loss, and MCC on the validation and test datasets for the nine colors with 11 angles were 0.919 ± 0.014 (ROC_AUC_valid), 0.895 ± 0.013 (ROC_AUC_test), 0.861 ± 0.023 (BAC_valid), 0.834 ± 0.013 (BAC_test), 0.515 ± 0.097 (PR_AUC_valid), 0.484 ± 0.085 (PR_AUC_test), 0.916 ± 0.018 (F_valid), 0.919 ± 0.015 (F_test), 0.881 ± 0.029 (Acc_valid), 0.886 ± 0.025 (Acc_test), 0.160 ± 0.078 (loss_train), 0.172 ± 0.066 (loss_valid), 0.156 ± 0.043 (loss_test), 0.360 ± 0.051 (MCC_valid), and 0.344 ± 0.034 (MCC_test) (Figure 12, right; Figures S15, S16, S19, S20, S22; Table S8). The highest prediction performance values among the nine colors on the validation and test datasets were 0.927 ± 0.019 for wheat (ROC_AUC_valid), 0.899 ± 0.019 for orange (ROC_AUC_test), 0.874 ± 0.026 for wheat (BAC_valid), 0.839 ± 0.017 for white (BAC_test), 0.581 ± 0.081 for white (PR_AUC_valid), 0.524 ± 0.084 for orange (PR_AUC_test), 0.930 ± 0.015 for wheat (F_valid), 0.924 ± 0.014 for white (F_test), 0.895 ± 0.023 for white (Acc_valid), 0.903 ± 0.024 for wheat (Acc_test), 0.400 ± 0.054 for wheat (MCC_valid), and 0.359 ± 0.038 for white (MCC_test) (Figure 12, right; Figures S18–S22; Table S8). The lowest prediction performance values on the training, validation, and test datasets for the nine colors were 0.122 ± 0.058 for yellow (loss_train), 0.129 ± 0.037 for white (loss_valid), and 0.141 ± 0.024 for wheat (loss_test) (Figure S22; Table S8).

**Figure 13:**
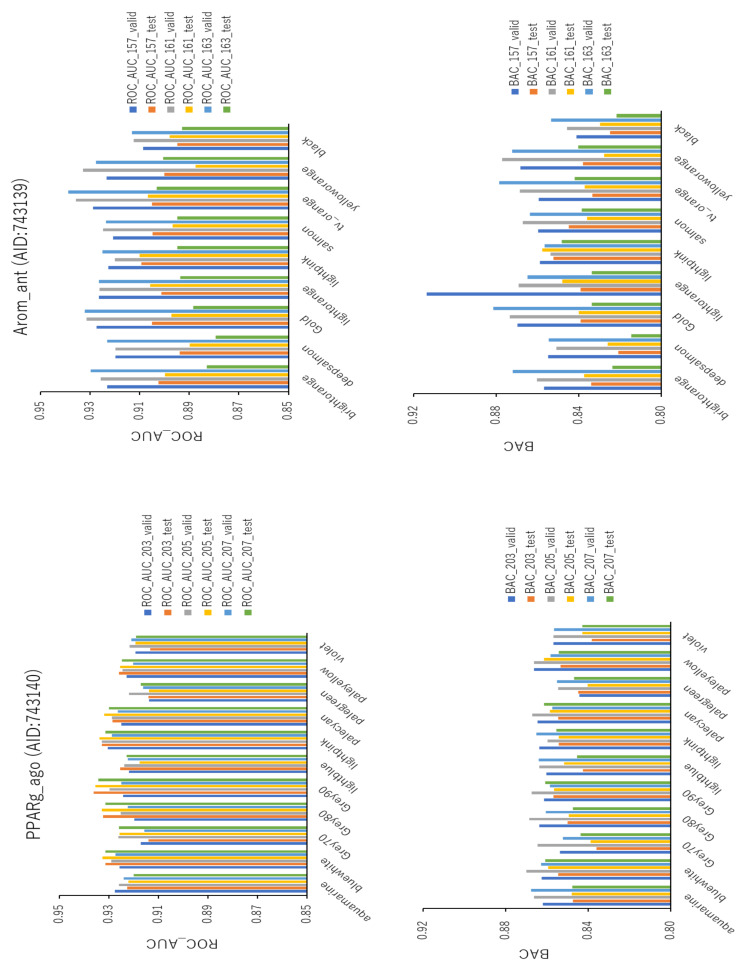
ROC_AUC and BAC for PPARg_ago (AID:743140) and Arom_ant (AID:743139) using TensorFlow with different background colors. Three-fold experiments were performed.

In addition, to study the effect of the background colors on the prediction performance of the improved DeepSnap-DL method in greater detail, we selected 11 additional colors (i.e., aquamarine (RGB:0.5,1.0,1.0), blue-white (RGB:0.85,0.85,1.00), Grey70 (RGB:0.7,0.7,0.7), Grey80 (RGB:0.8,0.8,0.8), Grey90 (RGB:0.9,0.9,0.9), light blue (RGB:0.75,0.75,1.00), light pink (RGB:1.00,0.75,0.87), pale cyan (RGB:0.8,1.0,1.0), pale green (RGB:0.65,0.90,0.65), pale yellow (RGB:1.0,1.0,0.5), and violet (RGB:1.0,0.5,1.0)) for the generation of images in DeepSnap with three angles (i.e., 203°, 205°, and 207°) using the validation and test datasets for PPARg_ago (Figure 13,left; Figures S23–S25, left; Table S9). The mean values of ROC_AUC, BAC, PR_AUC, F, Acc, loss, and MCC on the validation and test datasets for the 11 colors with three angles were 0.924 ± 0.004 (ROC_AUC_valid), 0.926 ± 0.007 (ROC_AUC_test), 0.861 ± 0.006 (BAC_valid), 0.850 ± 0.007 (BAC_test), 0.251 ± 0.026 (PR_AUC_valid), 0.339 ± 0.052 (PR_AUC_test), 0.887 ± 0.009 (F_valid), 0.874 ± 0.013 (F_test), 0.829 ± 0.014 (Acc_valid), 0.807 ± 0.020 (Acc_test), 0.207 ± 0.021 (loss_train), 0.184 ± 0.022 (loss_valid), 0.151 ± 0.025 (loss_test), 0.282 ± 0.010 (MCC_valid), and 0.263 ± 0.013 (MCC_test) (Figure 13, left; Figures S23–S25, left; Table S9). The highest prediction performance values among the 11 colors on the validation and test datasets were 0.931 ± 0.002 for light pink (ROC_AUC_valid), 0.935 ± 0.001 for Grey90 (ROC_AUC_test), 0.865 ± 0.003 for aquamarine (BAC_valid), 0.858 ± 0.003 for blue-white (BAC_test), 0.276 ± 0.022 for light pink (PR_AUC_valid), 0.413 ± 0.007 for Grey80 (PR_AUC_test), 0.897 ± 0.009 for Grey80 (F_valid), 0.884 ± 0.012 for light pink (F_test), 0.845 ± 0.015 for Grey80 (Acc_valid), 0.824 ± 0.020 for light pink (Acc_test), 0.295 ± 0.013 for Grey80 (MCC_valid), and 0.275 ± 0.015 for light pink (MCC_test) (Figure 13, left; Figures S23–S25, left; Table S9). The lowest prediction performance values on the training, validation, and test datasets for the 11 colors were 0.173 ± 0.019 for light pink (loss_train), 0.170 ± 0.023 for Grey80 (loss_valid), and 0.129 ± 0.008 for Grey90 (loss_test) (Figure S25, left; Table S9).

In addition, we used nine colors (i.e., bright orange (RGB:1.0,0.7,0.2), deep salmon (RGB:1.0,0.5,0.5), gold (RGB:1.00,0.82,0.14), light orange (RGB:1.0,0.8,0.5), light pink (RGB:1.00,0.75,0.87), salmon (RGB:1.0,0.6,0.6), tv_orange (RGB:1.00,0.55,0.15), yellow-orange (RGB:1.00,0.87,0.37), and black (RGB:0,0,0)) to generate images using the improved DeepSnap-DL method with three angles (i.e., 157°, 161°, and 163°) on the validation and test datasets for Arom_ant (Figure 13, left; Figures S23–S25, right; Table S10).

The mean values of ROC_AUC, BAC, PR_AUC, F, Acc, loss, and MCC on the validation and test datasets for the 11 colors with three angles were 0.926 ± 0.006 (ROC_AUC_valid), 0.898 ± 0.005 (ROC_AUC_test), 0.865 ± 0.014 (BAC_valid), 0.836 ± 0.010 (BAC_test), 0.547 ± 0.046 (PR_AUC_valid), 0.487 ± 0.059 (PR_AUC_test), 0.918 ± 0.014 (F_valid), 0.913 ± 0.014 (F_test), 0.884 ± 0.023 (Acc_valid), 0.877 ± 0.023 (Acc_test), 0.151 ± 0.044 (loss_train), 0.136 ± 0.034 (loss_valid), 0.140 ± 0.018 (loss_test), 0.365 ± 0.033 (MCC_valid), and 0.332 ± 0.031 (MCC_test) (Figure 13, left; Figures S23–S25, right; Table S10). The highest prediction performance values among the 11 colors on the validation and test datasets were 0.934 ± 0.005 for tv_orange (ROC_AUC_valid), 0.905 ± 0.002 for tv_orange (ROC_AUC_test), 0.883 ± 0.027 for light orange (BAC_valid), 0.853 ± 0.005 for light pink (BAC_test), 0.614 ± 0.011 for light orange (PR_AUC_valid), 0.553 ± 0.041 for light pink (PR_AUC_test), 0.935 ± 0.009 for light orange (F_valid), 0.938 ± 0.004 for light pink (F_test), 0.911 ± 0.015 for light orange (Acc_valid), 0.918 ± 0.007 for light pink (Acc_test), 0.420 ± 0.012 for light orange (MCC_valid), and 0.404 ± 0.010 for light pink (MCC_test) (Figure 13, left; Figures S23–S25, right; Table S10). The lowest prediction performance values on the training, validation, and test datasets for the 11 colors were 0.110 ± 0.047 for light orange (loss_train), 0.107 ± 0.037 for light pink (loss_valid), and 0.120 ± 0.001 for salmon (loss_test) (Figure S25, right; Table S10). 

## 3. Discussion

In this study, we analyzed the effects of angles on the generation of images using two DeepSnap-DL systems, one with TensorFlow and Keras and the other with DIGITS. In each case, we used two MIEs, PPARg_ago (AID:743140) and Arom_ant (AID:743139). In the prediction models constructed using DeepSnap-DL with DIGITS, the prediction performance increased as the number of angles decreased in DeepSnap, which can use various angles, resulting in a larger number of images generated by DeepSnap. By contrast, we observed two peaks—around two angles of 180° and 360°—in the prediction performance of the models constructed using TensorFlow and Keras. In addition, the prediction performance of DeepSnap-DL using TensorFlow and Keras decreased markedly as the number of angles used in DeepSnap decreased.

It has recently been reported that convolutional neural networks (CNNs) are effective in dealing with the movement of objects, but they are not suitable for viewpoint conversions such as rotation and scaling because they cannot understand the relationships between parts within the image [44,45,46,47,48,49]. Judging from these reports, there may be angles in DeepSnap for which the relative positional relationships of the features extracted with each angle cannot be understood. Therefore, clarifying appropriate depiction angles in DeepSnap may be an important factor for practical use. In addition, there are still some problems with the extraction of features using CNNs, that is, “a black box problem”, whose problem makes it difficult to identify the region in the image extracted automatically as a feature by CNNs. Therefore, in order to estimate the area in the image extracted as a feature by the CNNs in this DeepSnap-DL system, the gradient with respect to the predicted value is weighted using Gradient-weighted Class Activation Mapping (GRAD-CAM) for the visualization of important pixels [50]. As a result, the chemical structure part within the image generated by DeepSnap was detected as the feature by the GRAD-CAM (data not shown). Thus, this finding suggests that the DeepSnap-DL system recognized and classified based on the stereochemical structure in the image used as input data. 

In addition, we used a loss-evaluation index to examine the contributions of the LRs and BSs to the prediction performance of DeepSnap-DL using TensorFlow and Keras, as the loss-evaluation index represents the difference between the output of the neural network and the data from the training, validation, and test datasets of the two MIEs, PPARg_ago (AID:743140) and Arom_ant (AID:743139). During training on these datasets, the goal of the neural network was to reduce the loss value on the training dataset. Overfitting—which is the deviation of the loss values among the training, validation, and test datasets during learning—can be prevented by adjusting the parameters, such as the LRs and BSs. In this study, we demonstrated that optimization of these parameters can prevent discrepancies between the prediction performance on the training, validation, and test datasets.

In addition, we investigated the effects of the background colors of the images generated by DeepSnap-DL using two MIEs, PPARg_ago (AID:743140) and Arom_ant (AID:743139). Previously, we reported that using black and white as background colors led to a slightly lower performance of the prediction models constructed using the DeepSnap-DL DIGITS system, as compared with four other background colors (i.e., red, yellow, green, and blue) [41]. In the present study, the prediction model for PPARg_ago (AID:743140) constructed by DeepSnap-DL using TensorFlow and Keras exhibited higher performance for three background colors (i.e., wheat, white, and yellow) than for six other background colors (i.e., blue, cyan, green, magenta, orange, and red). Furthermore, the prediction model for Arom_ant (AID:743139) constructed by DeepSnap-DL using TensorFlow and Keras exhibited higher performance for three background colors (i.e., wheat, white, and orange) than for six other background colors (i.e., blue, cyan, green, magenta, yellow, and red). These differences in the effect of the background colors on the prediction performance of DeepSnap-DL using TensorFlow and Keras and of the DeepSnap-DL DIGITS system may be due to the different background colors used in the pre-training. Our results suggest that the improved DeepSnap-DL system has the potential to improve the prediction performance further by optimizing the aforementioned parameters. In the future, it would be a useful chemoinfomatics tool when the improved system is shaped into an internet server or computer program.

## 4. Materials and Methods

### 4.1. Data

We downloaded information related to the input data—including the chemical structures in SMILES format and the corresponding agonist or antagonist scores, defined as Pubchem_activity_scores—from the Tox21 10K library in the PubChem database, as previously reported [38,39,40,41] (Table S11). After eliminating overlapping chemicals, we determined the agonist and antagonist scores in the range from 0% to 100% by normalizing each titration point relative to the positive control compound and dimethyl sulfoxide (DMSO)-only wells according to the following equation: % activity = [(Vcompound − Vdmso)/(Vpos − Vdmso)] × 100, where Vcompound, Vdmso, and Vpos denote the compound-well values, the median values of the DMSO-only wells, and the median value of the positive control well in the reporter gene assay, respectively. The scores were then grouped into three classes, namely (i) 0, (ii) 1–39, and (iii) 40–100, which represent inactive, inconclusive, and active compounds, respectively. In this study, compounds with activity scores of 40–100 and 0–39 were defined as active and inactive, respectively, in all datasets. We downloaded information from the bioassay on 59 MIEs from the Tox21 10K library in PubChem [51] (Tables S11–S14). 

### 4.2. DeepSnap

We applied the SMILES format for 3D conformational import using the SMILES_TO_SDF program, which built 3D chemical structure from SMILES strings containing the steric conformation information of chemical structures to generate a chemical database saved in SDF format in the DeepSnap-DL system, as previously reported [37,38,39,40,41]. Using the SDF files prepared by SMILES_TO_SDF, 3D chemical structures were depicted as 3D ball-and-stick models with different colors corresponding to different atoms. We used PyMOL, an open-source molecular visualization system written in the Python programming language (Schrödinger, Inc., New York, NY, US) to obtain high-quality 3D molecular modeling of the chemical structures. The 3D chemical structures can produce different images depending on the direction, and they are captured automatically by DeepSnap as snapshots with user-defined angle increments with respect to the *x*, *y*, and *z* axes. The snapshots, saved as 256 × 256-pixel PNG files (RGB), were divided into three datasets: training, validation, and test. Additionally, the external test dataset is permanently fixed. All two-dimensional PNG images produced by the modified DeepSnap system were used for training and fine-tuning the prediction models using TensorFlow and Keras on CentOS Linux 7.3.1611 with the convolutional neural network GoogLeNet. Background colors in the images were changed to the color values in PyMOL [52].

### 4.3. Evaluation of Model Quality

The DeepSnap-DL system automatically obtains the probability of prediction results with the lowest minimum loss_valid value among 30 examined epochs, which are the numbers of repeats for one training dataset modulated by early stopping. In addition, the performance of each model was automatically calculated in terms of the metrics ROC_AUC, PR_AUC, BAC, F, MCC, Acc, and loss. These performance metrics are defined as follows, where TP, FN, TN, and FP denote true positive, false negative, true negative, and false positive, respectively.

Sensitivity = Number of TPs (Number of TPs + Number of FNs)

= Number of TPs/Number of real positive in the data

Specificity = Number of TNs/(Number of TNs + Number of FPs)

= Number of TNs/Number of real negative in the data

BAC = Balanced accuracy = (sensitivity + specificity)/2

Acc = Accuracy = (Number of TP + Number of TN)/(Number of TP + Number of FP

+ Number of TN + Number of FN)

Precision = Number of TP/(Number of TP + Number of FP)

Recall = Number of TP/(Number of TP + Number of FN)

F-measure (F) = 2 × Recall × Precision/(Recall + Precision)

MCC = (TP ×TN − FP ×FN)/ $\sqrt{k}$, where MCC is the Matthews correlation coefficient and k = (TP + FP) × (TP + FN) × (TN + FP) × (TN + FN). To determine the optimal cutoff point for the definition of TP, FN, TN, and FP, we adopted a method of maximizing the sensitivity (1−specificity), called the Youden index. This index has a value ranging from 0 to 1, where 1 represents maximum effectiveness and 0 represents minimum effectiveness. In addition, the area under the curve (AUC) for the receiver operating characteristics (ROC) is given by

$$\mathrm{ROC}\_\mathrm{AUC}\;=\;1/Np\sum_{j=1}^{Np}f\left(j\right)$$

$$f(j)\;=\;1/T\sum_{t=1}^{T}Wt\left\{\begin{array}{c} 1\;\mathrm{if}\;pj≧t\\ 0\;\mathrm{otherwise}\; \end{array}\right\}$$

*Wt* = 1/2 (prec*_t_*_+1_
− prec*_t_*−_1_)

$${\mathrm{prec}}_{t}\;=\;\left(\#\;\mathrm{of}\;\mathrm{points}\;i\;\mathrm{where}\;\mathrm{pi}\;≧t\;\mathrm{and}\;ci=1\right)/\left(\#\;\mathrm{of}\;\mathrm{points}\;i\;\mathrm{where}\;\mathrm{pi}\;≧t\right)$$

Here, ROC_AUC denotes the area under the curve *f*, *j* iterates over the true points, *Np* is the number of true points, *T* is the number of thresholds, and prec*t* is the precision at threshold *t*. For broader cases, let prec0 = prec1, and precT = 0 [53].

The PR curve is plot of Recall (x) vs. Precision (y), and PR_AUC was calculated as reported previously [54]. This study used *N* = 3 to reduce the bias, and the values are represented as averages.

## 5. Conclusion

In this study, we used the Tox21 10K library to construct 59 prediction models for agonists and antagonists of MIEs in the AOP using an improved DeepSnap-DL system with TensorFlow and Keras, and we demonstrated that the prediction performance can be improved by optimizing various parameters. In addition, we improved the throughput of the previously reported DeepSnap-DL system by combining several steps into one. Thus, the improved DeepSnap-DL system may be a useful tool for in silico prediction of chemical compounds with high-performance and throughput in the AOP.

## Data Availability

We are applying all data requirements.

## Supplementary Materials

The following are available online at https://www.mdpi.com/article/10.3390/ijms221910821/s1,
