# Supplementary material for: Prediction Models for Agonists and Antagonists of Molecular Initiation Events for Toxicity Pathways Using an Improved Deep-Learning-Based Quantitative Structure–Activity Relationship System
Source: Int J Mol Sci. 2021 Oct 6;22(19):10821. doi: 10.3390/ijms221910821 (PMC8509615; doi:10.3390/ijms221910821)
Supplement: Supplementary file 1 [file ijms-22-10821-s001.zip › ijms-1378868-supplementary figures revised 3-done.pdf]

International Journal of
Molecular Sciences

MDPI

Supplementary materials

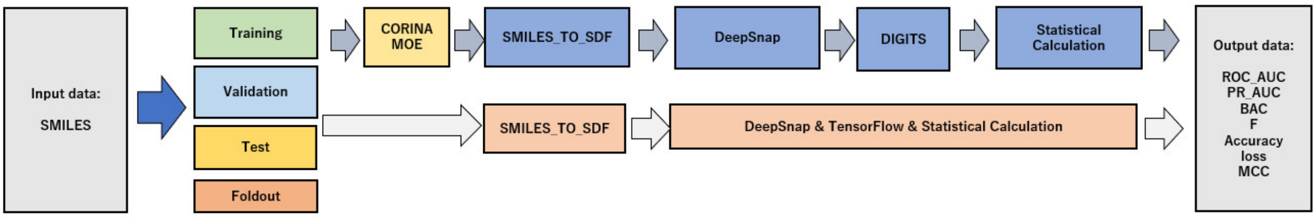

Figure S1. DeepSnap-DL systems. SMILES format in chemical compounds was used as input data for the DeepSnap, which was divided four groups into Training, Validation, Test, and Foldout datasets. In previous system, 3D conformational import from the SMILES format was performed by MOE 208 and CORINA classic software to generate the chemical database and then the 3D chemical structures were captured automatically as snapshots, named DeepSnap (upper line). And then, DL was performed using the snapshots as input data. Finally, prediction performance was evaluated by calculation of ROC_AUC, BAC, F, Accuracy, loss, and MCC. On the other hand, in this novel system, the generation of snapshots from the SMILES format was performed as one-step automatically. And then, DeepSnap, DL by TensorFlow, and statistical calculation of ROC_AUC, PR_AUC, BAC, F, Accuracy, loss, and MCC were executed sequentially by one-step.

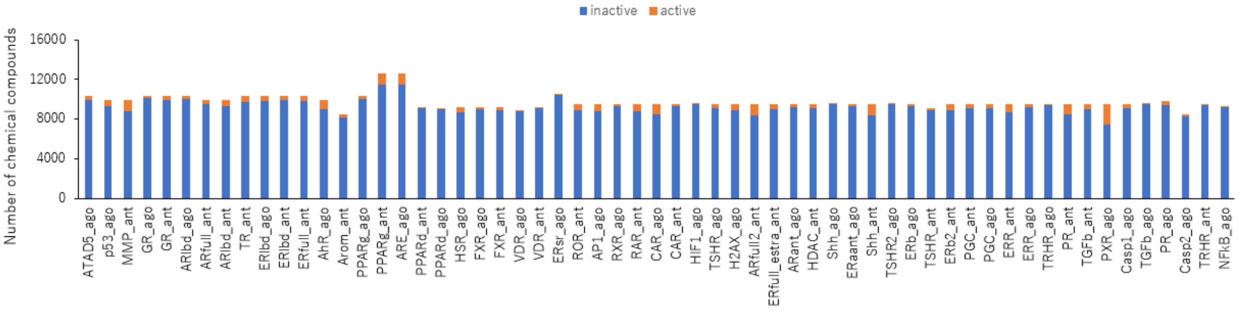

(**a**)

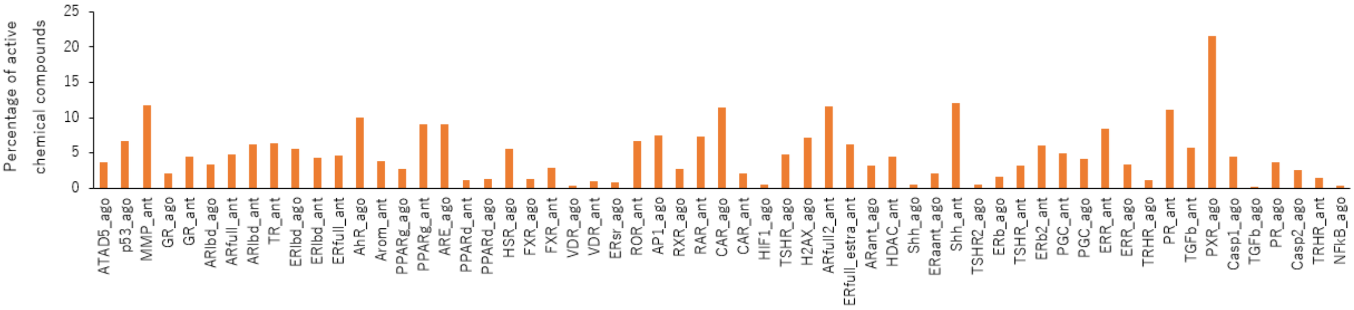

(**b**)

Figure S2. Number of chemical compounds used construction of prediction models of 59 MIEs with agonist or antagonist activities derived from the Tox21 10K library (**a**). Rate of active chemical compounds in total compounds (**b**).

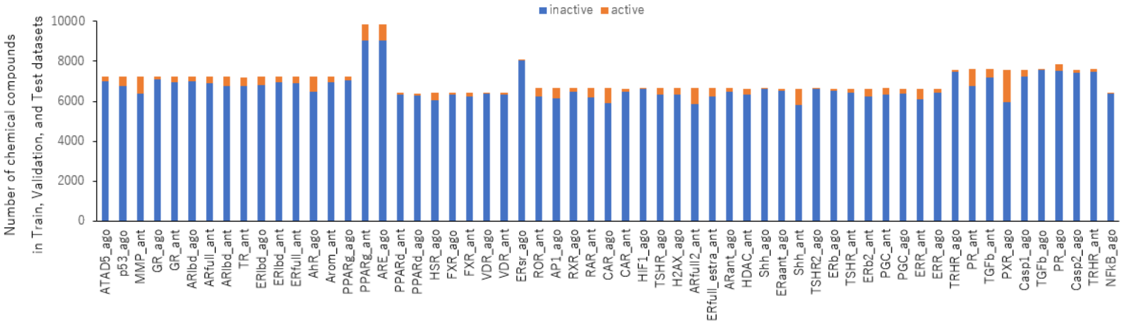

(**a**)

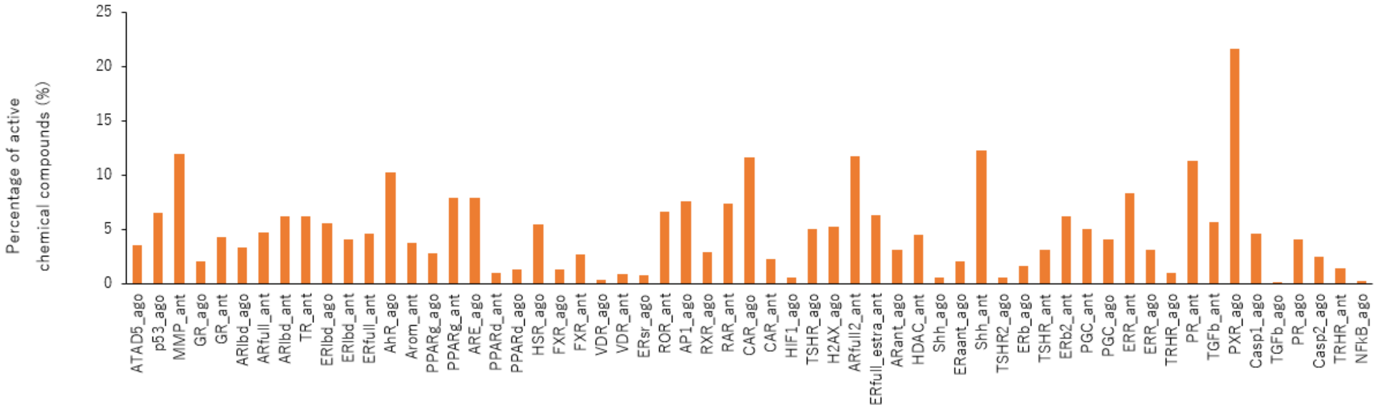

(**b**)

Figure S3. Number of chemical compounds of train, validation, and test datasets used construction of prediction models of 59 MIEs with agonist or antagonist activities derived from the Tox21 10K library (**a**). Percentage of active chemical compounds of train, validation, and test datasets in total compounds (**b**).

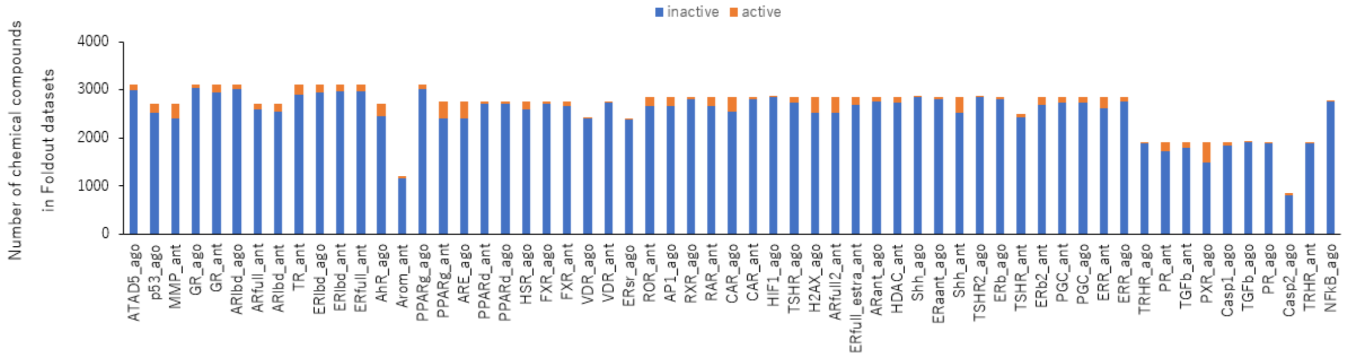

(**a**)

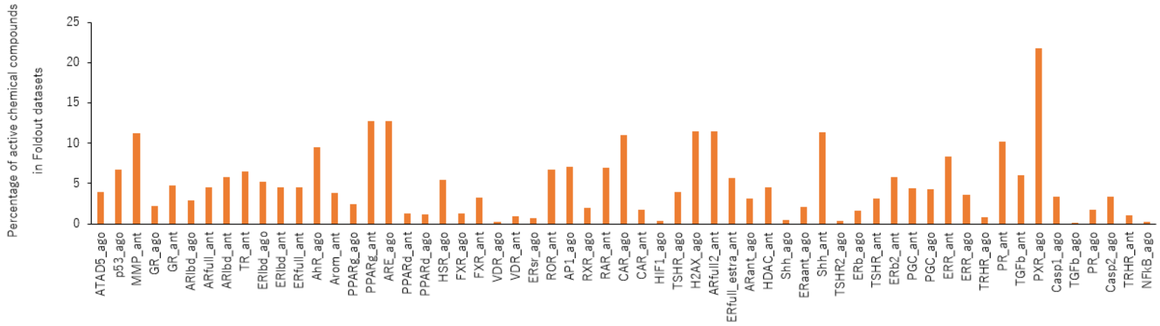

(**b**)

Figure S4. Number of chemical compounds of Foldout dataset used construction of prediction models of 59 MIEs with agonist or antagonist activities derived from the Tox21 10K library (**a**). Rate of active chemical compounds of Foldout dataset in total compounds (**b**).

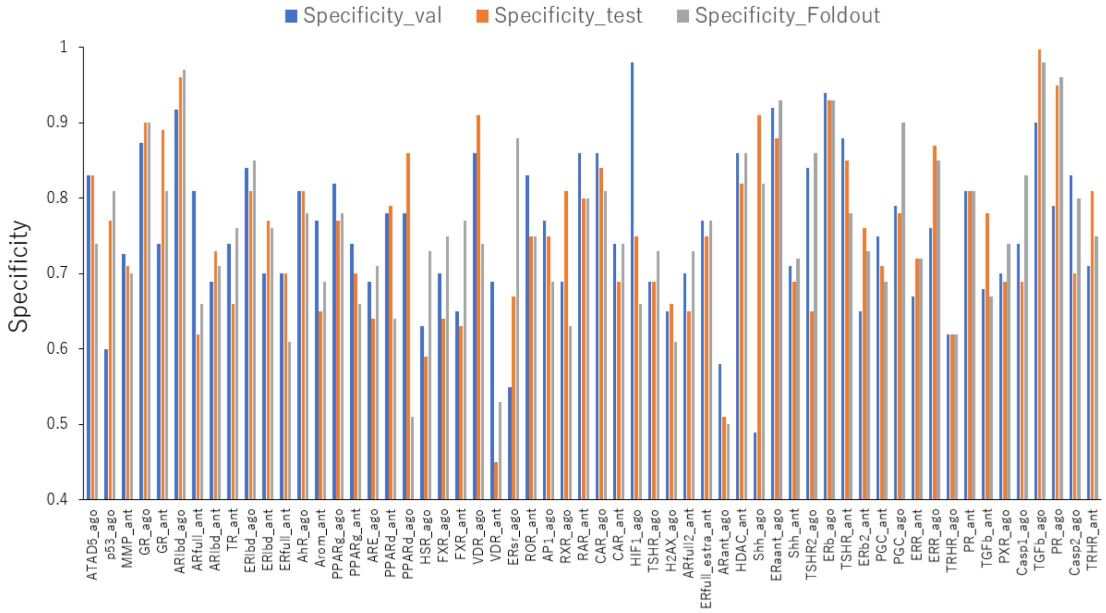

Figure S5. Specificity in valid, test, and foldout datasets. Among six angles analyzed in the DeepSnap of this study, most high performances were indicated.

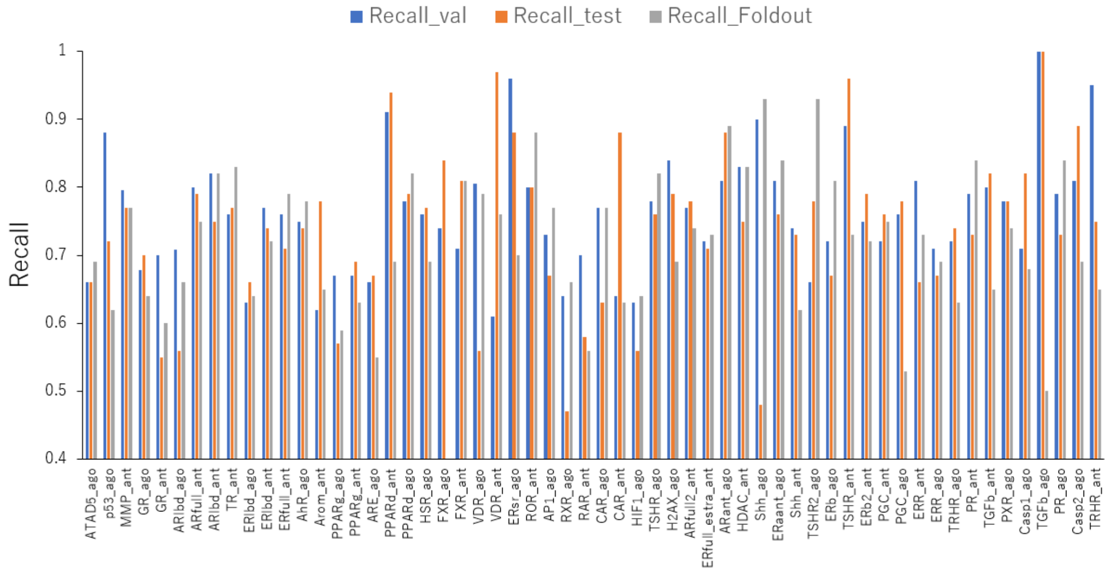

Figure S6. Recall in valid, test, and foldout datasets. Among six angles analyzed in the DeepSnap of this study, most high performances were indicated.

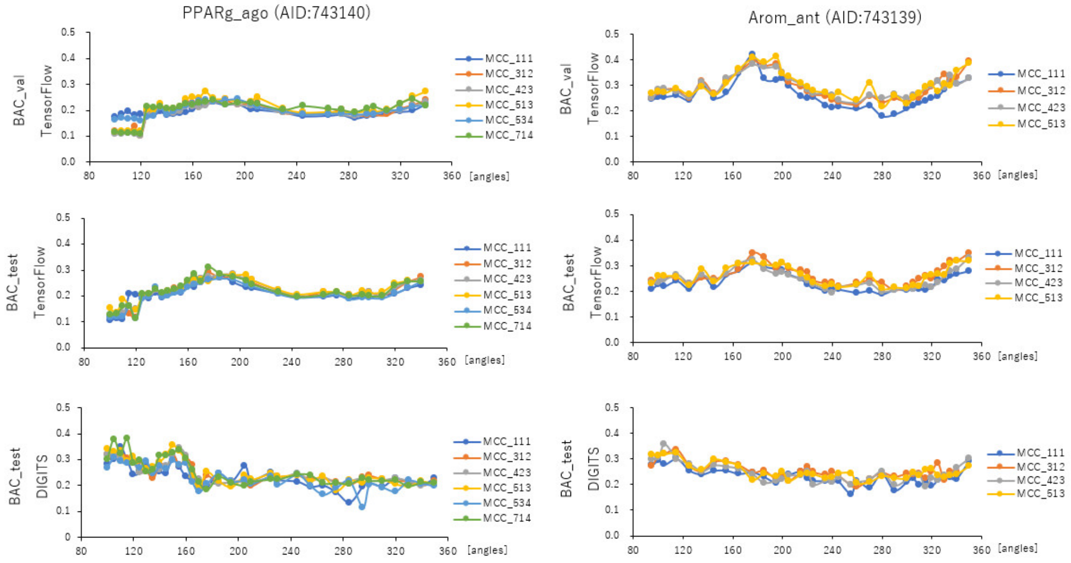

Figure S7. MCC of PPARg_ago (AID:743140) and Arom_ant (AID:743139) in valid and test datasets by TensorFlow and test dataset by DIGITS with 31 angles in DeepSnap.

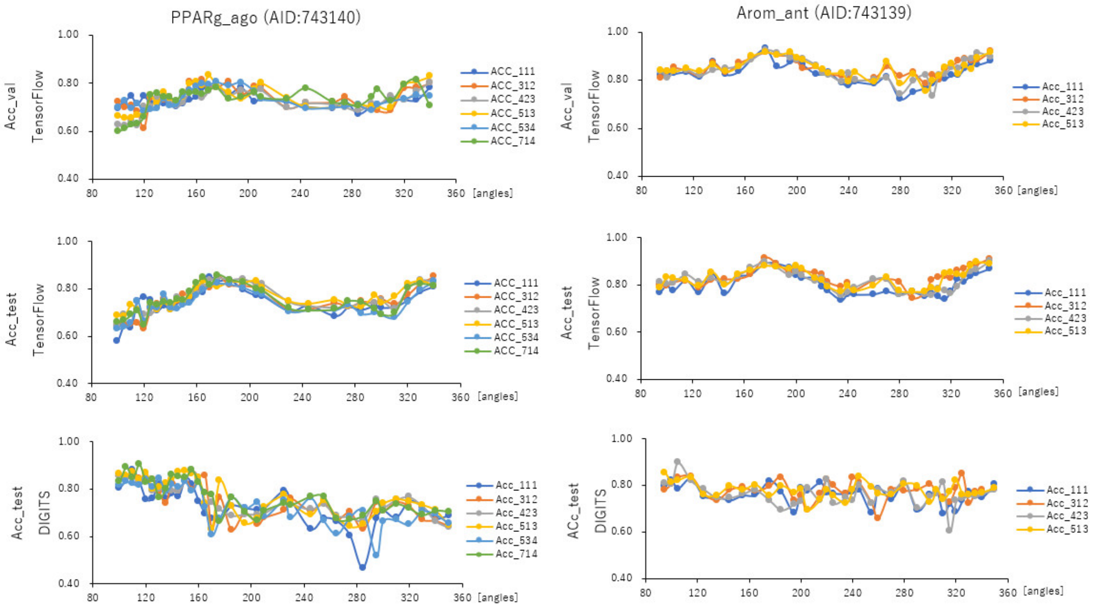

Figure S8. Acc of PPARg_ago (AID:743140) and Arom_ant (AID:743139) in valid and test datasets by TensorFlow and test dataset by DIGITS with 31 angles in DeepSnap.

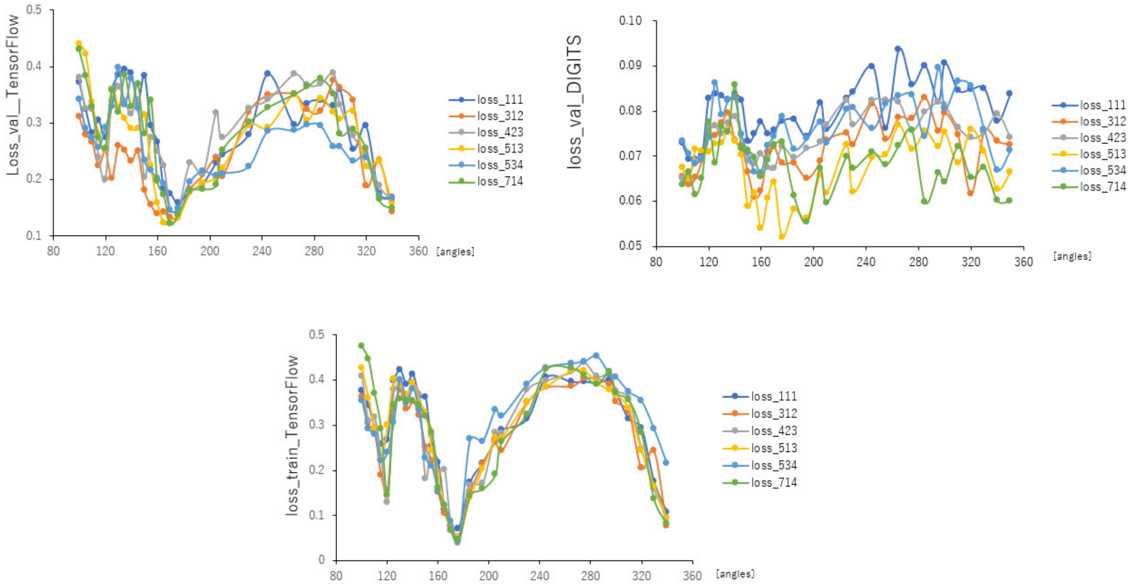

Figure S9. loss of PPARg_ago (AID:743140) in train and valid datasets by TensorFlow and valid dataset by DIGITS with angles in DeepSnap.

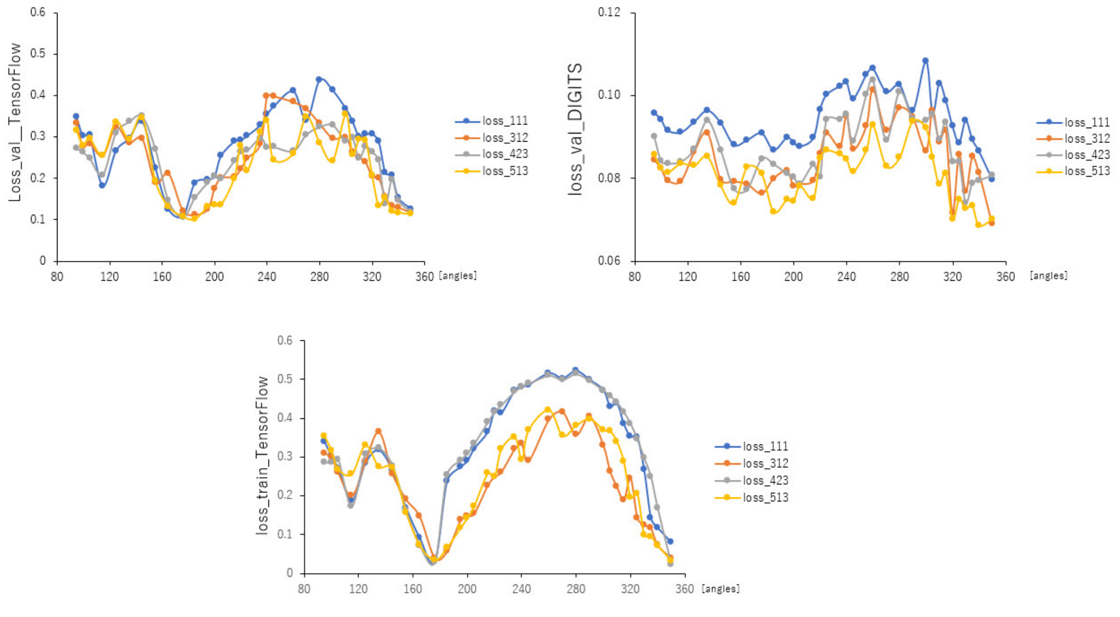

Figure S10. loss of Arom_ant (AID:743139) in train and valid datasets by TensorFlow and valid dataset by DIGITS with angles in DeepSnap.

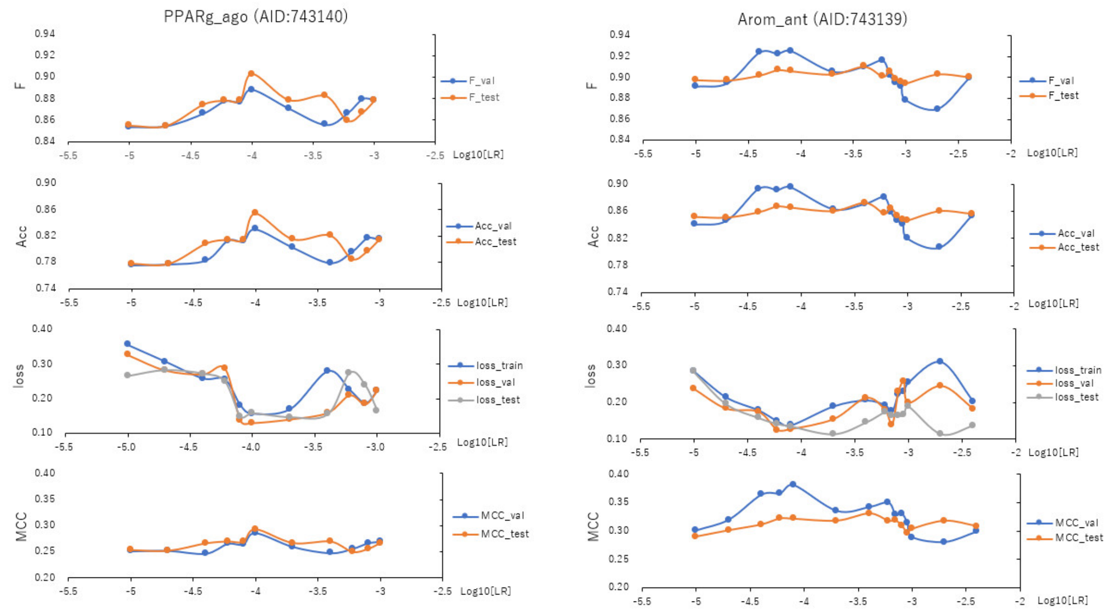

Figure S11. F, Acc, loss, and MCC of PPARg_ago (AID:743140) and Arom_ant (AID:743139) by TensorFlow with learning rate (LR).

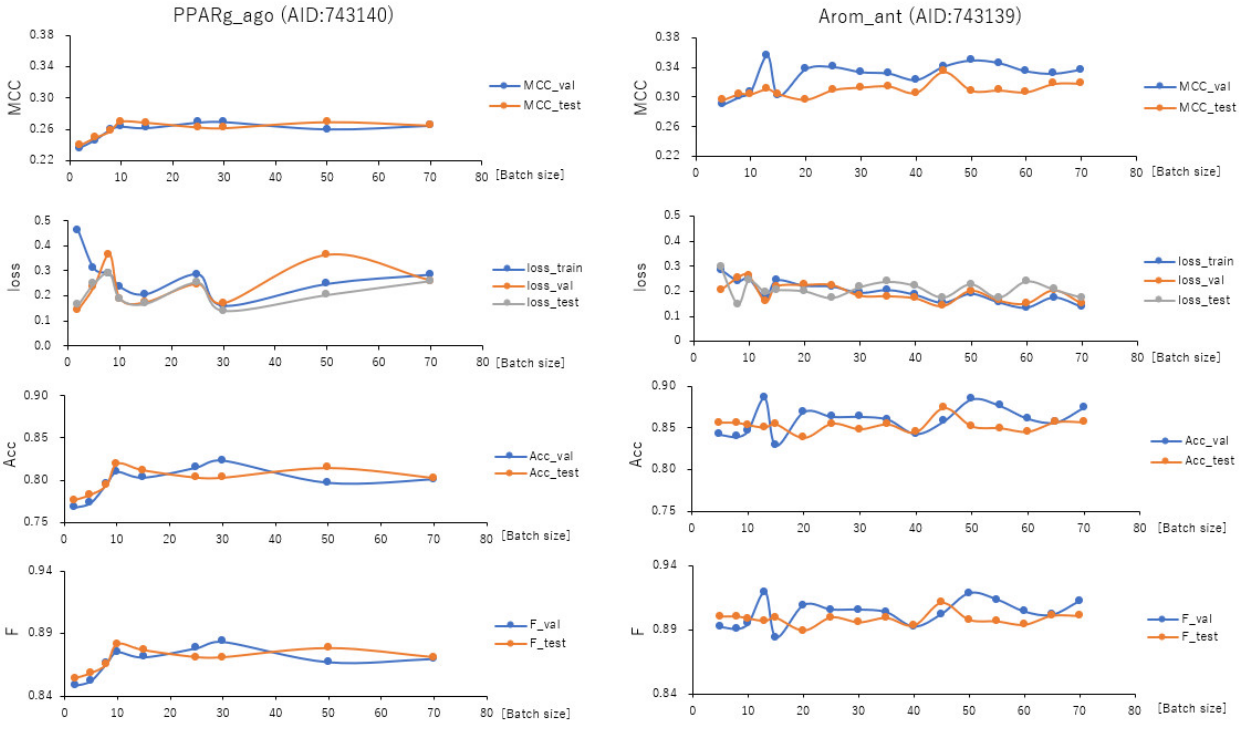

Figure S12. MCC, loss, Acc, and F of PPARg_ago (AID:743140) and Arom_ant (AID:743139) by TensorFlow with batch size.

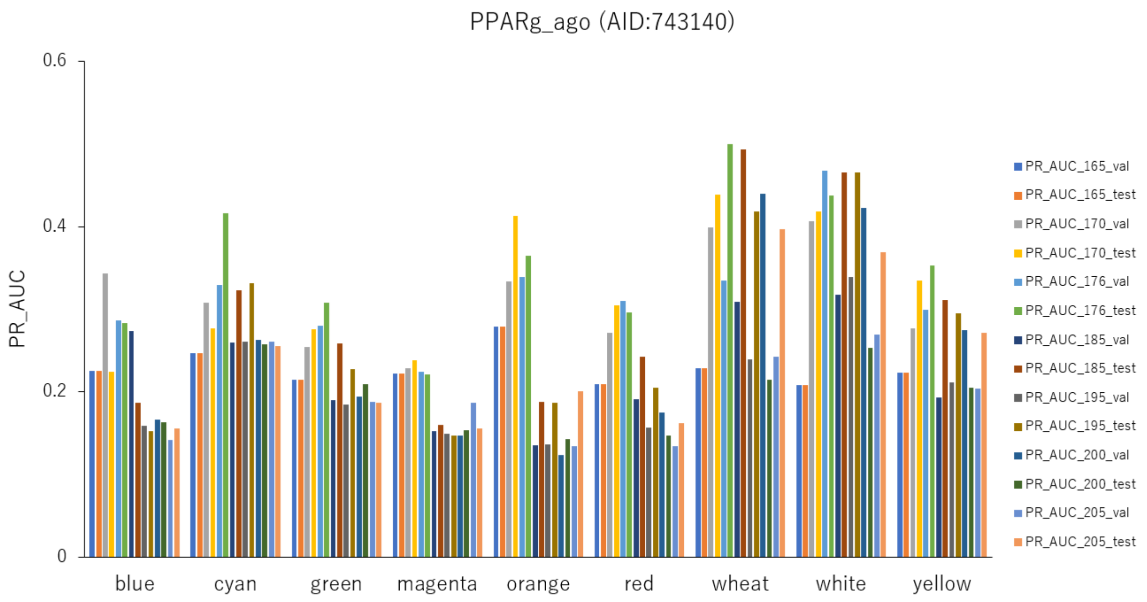

Figure S13. PR_AUC of PPARg_ago (AID:743140) by TensorFlow with background colors.

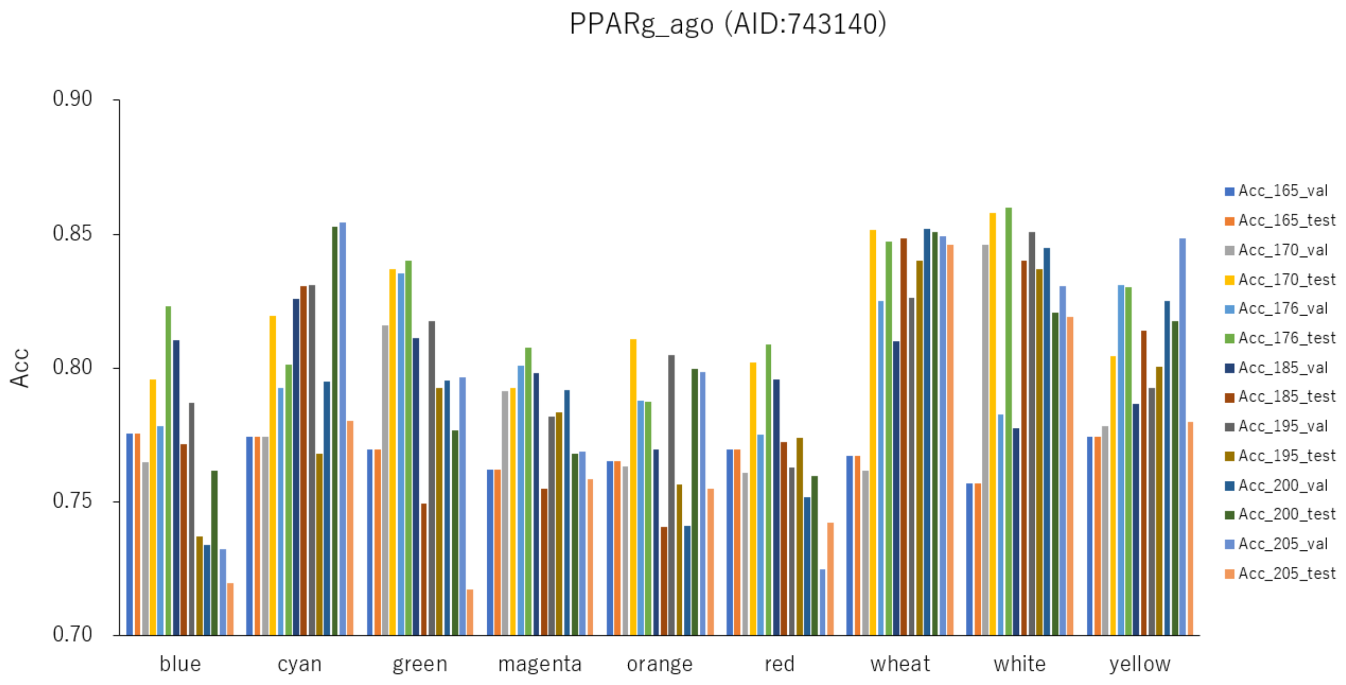

Figure S14. Acc of PPARg_ago (AID:743140) by TensorFlow with background colors.

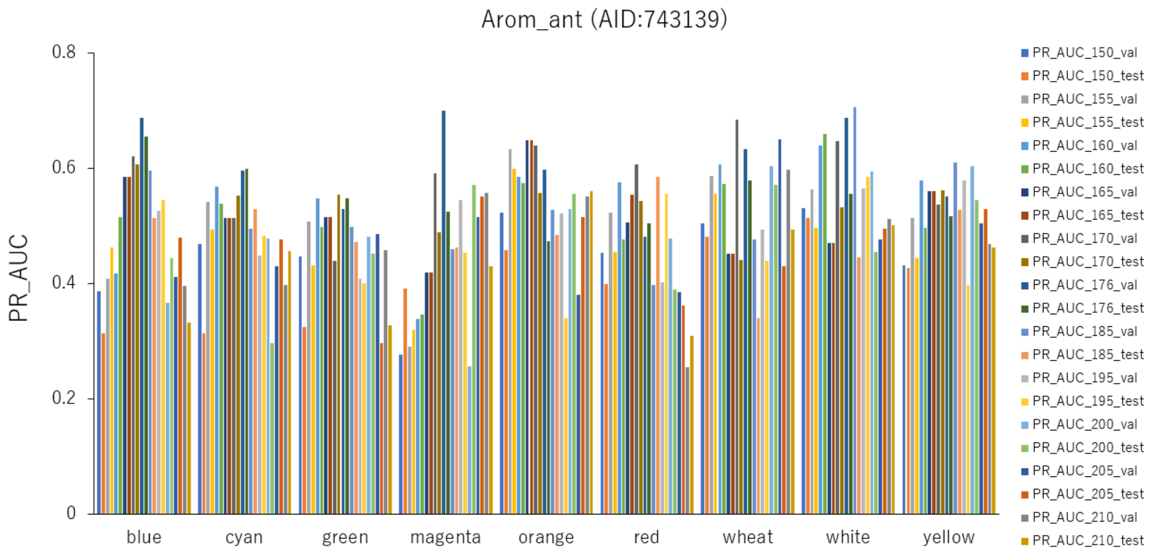

Figure S15. PR_AUC of Arom_ant (AID:743139) by TensorFlow with background colors.

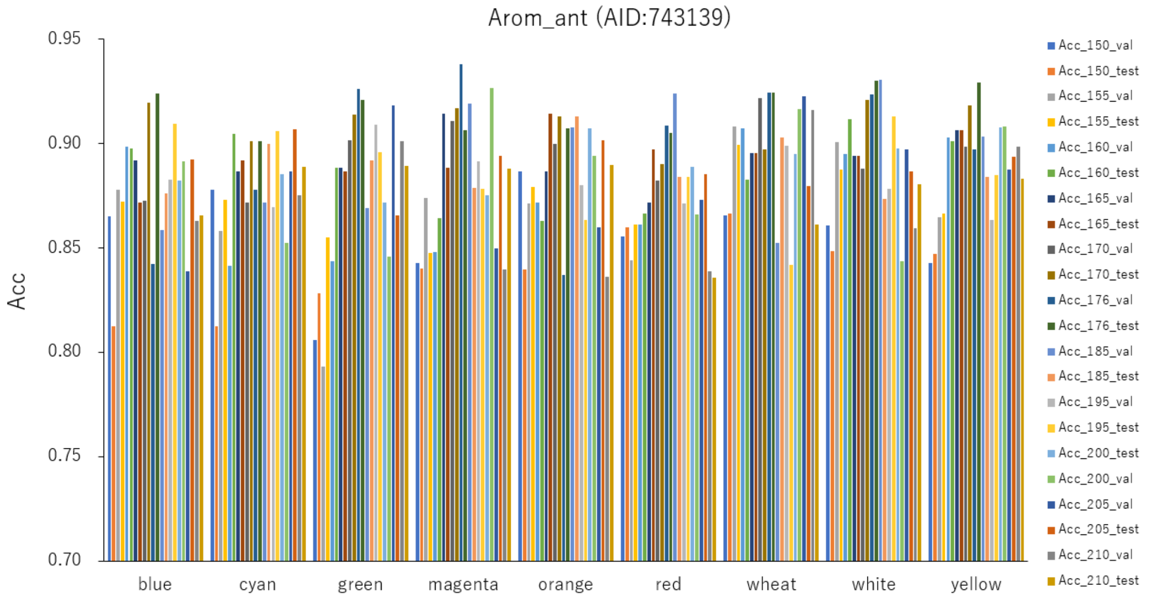

Figure S16. Acc of Arom_ant (AID:743139) by TensorFlow with background colors.

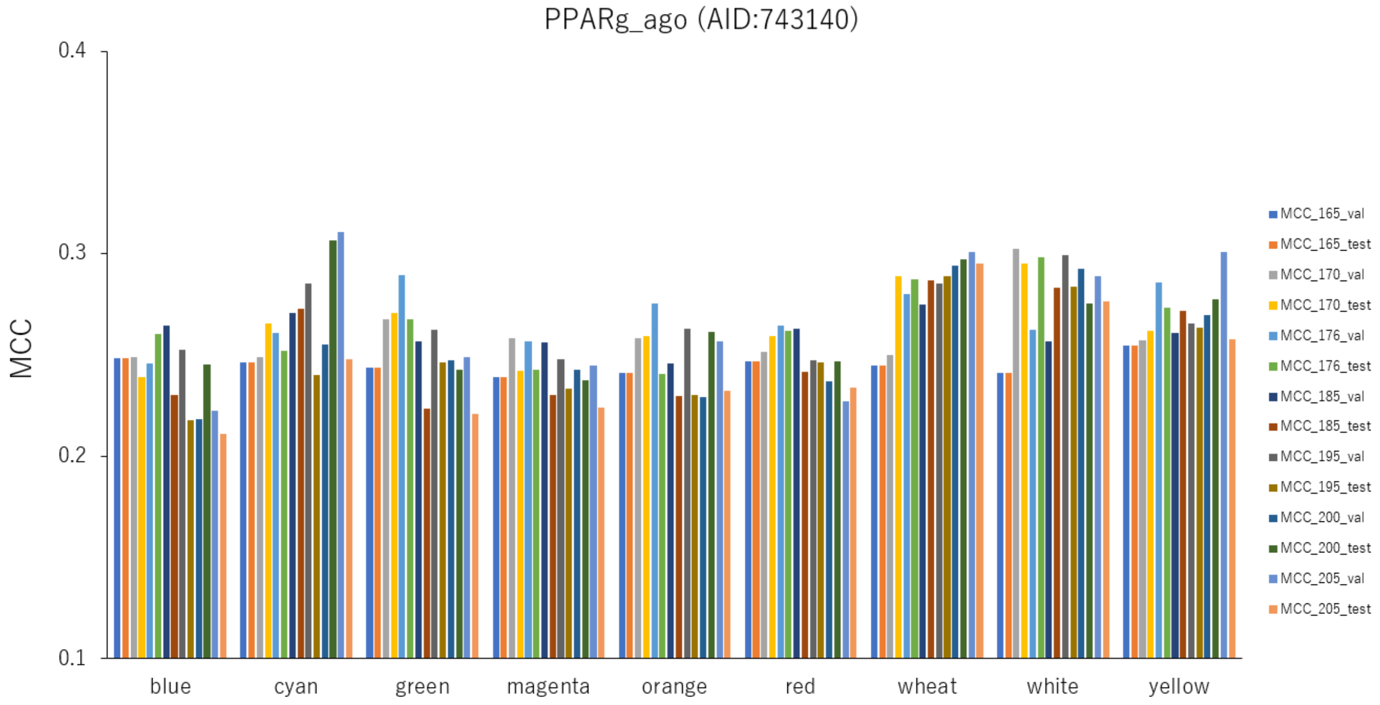

Figure S17. MCC of PPARg_ago (AID:743140) by TensorFlow with background colors.

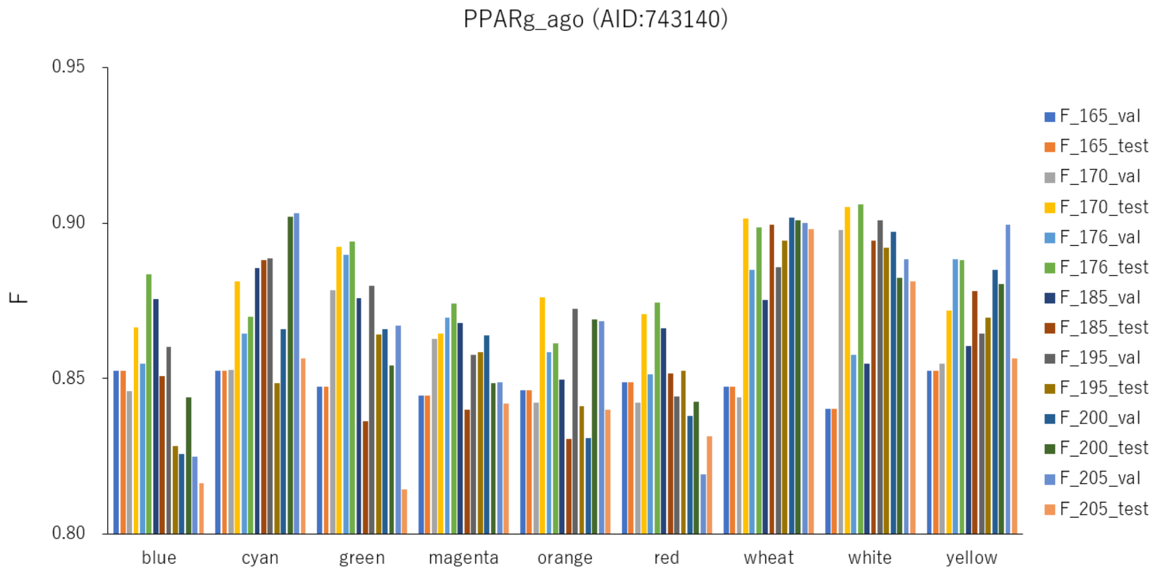

Figure S18. F of PPARg_ago (AID:743140) by TensorFlow with background colors.

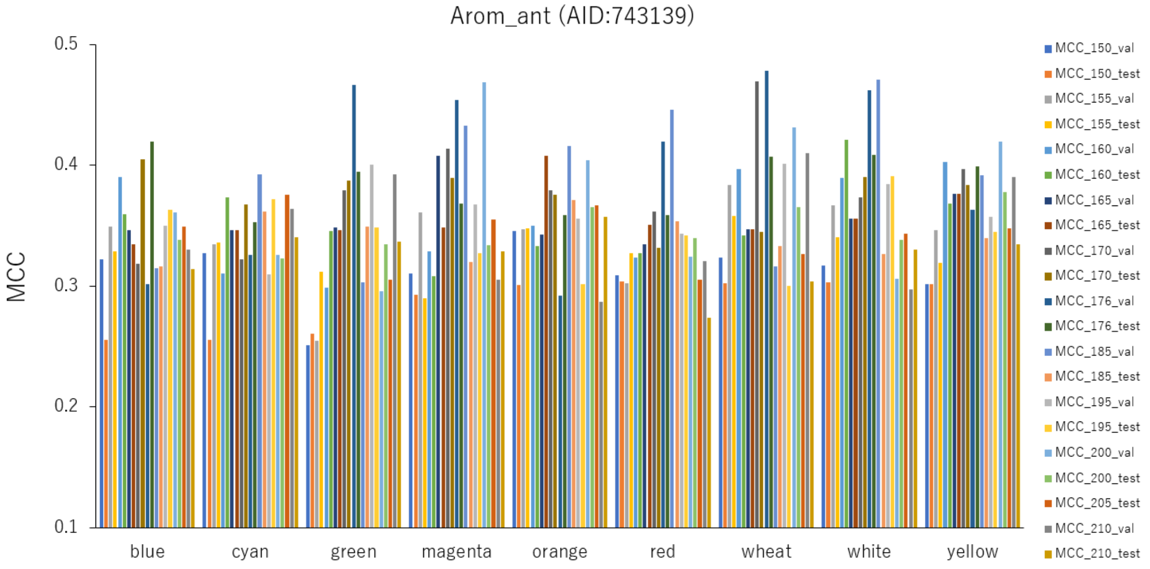

Figure S19. MCC of Arom_ant (AID:743139) by TensorFlow with background colors.

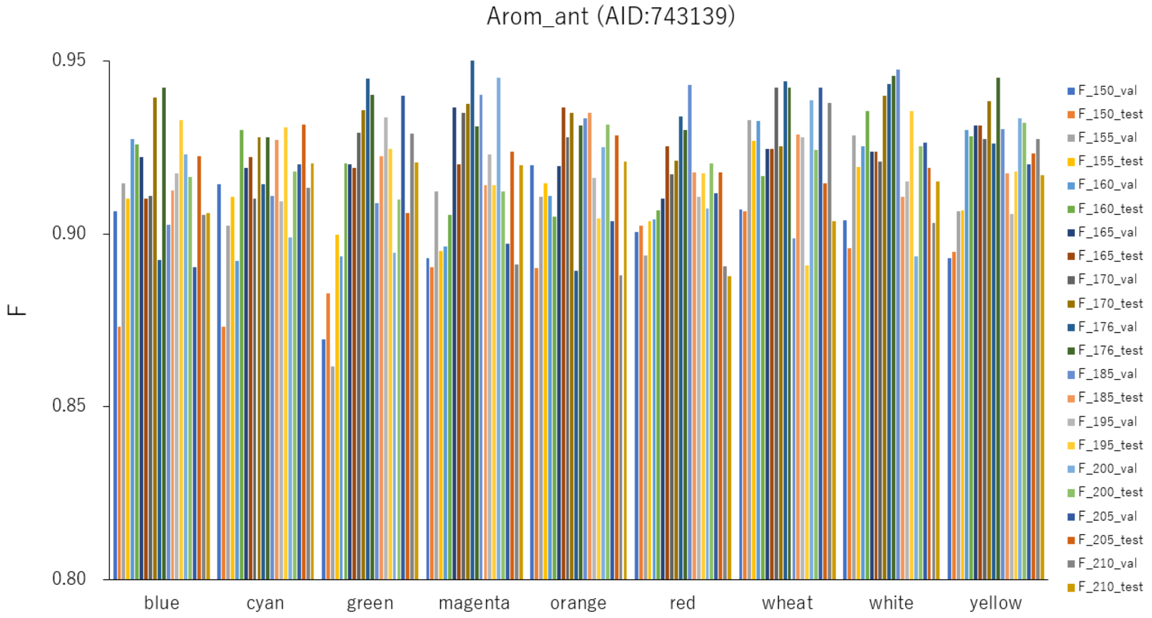

Figure S20. F of Arom_ant (AID:743139) by TensorFlow with background colors.

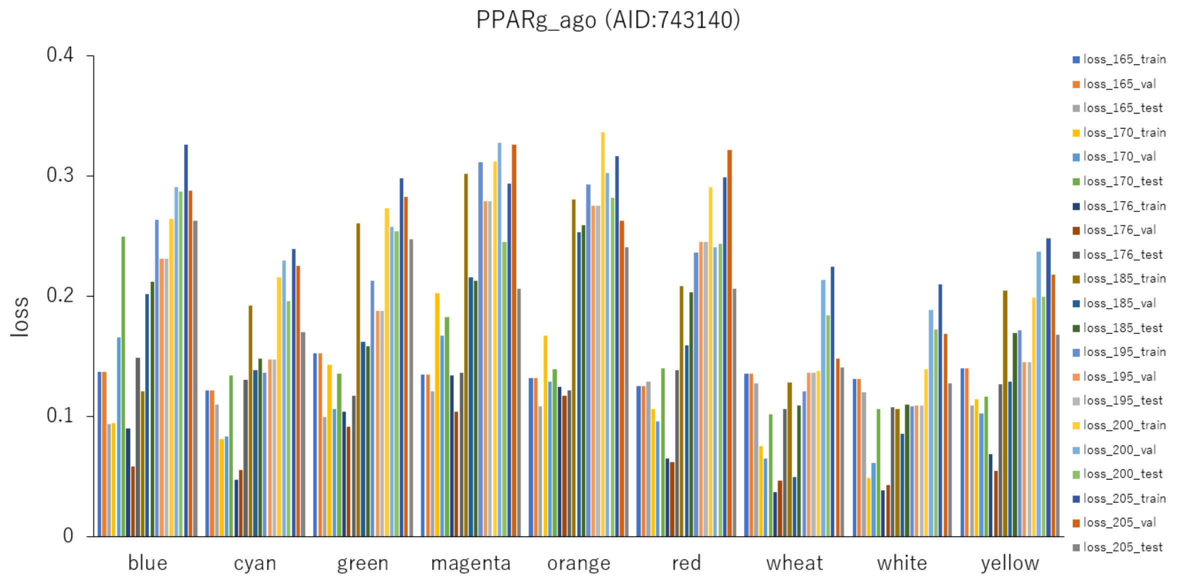

Figure S21. loss of PPARg_ago (AID:743140) by TensorFlow with background colors.

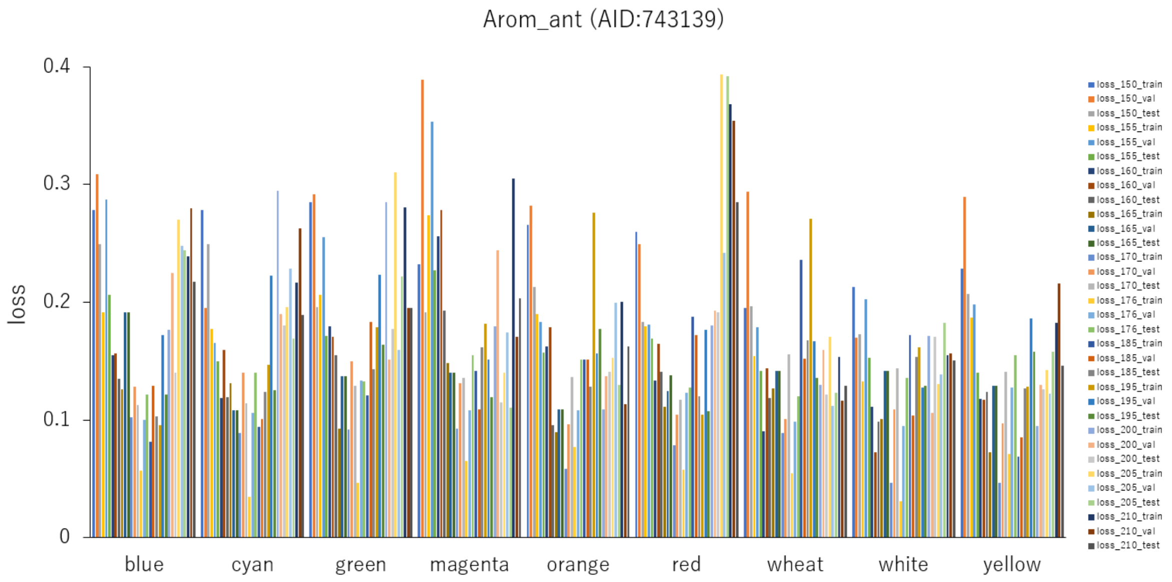

Figure S22. loss of Arom_ant (AID:743139) by TensorFlow with background colors.

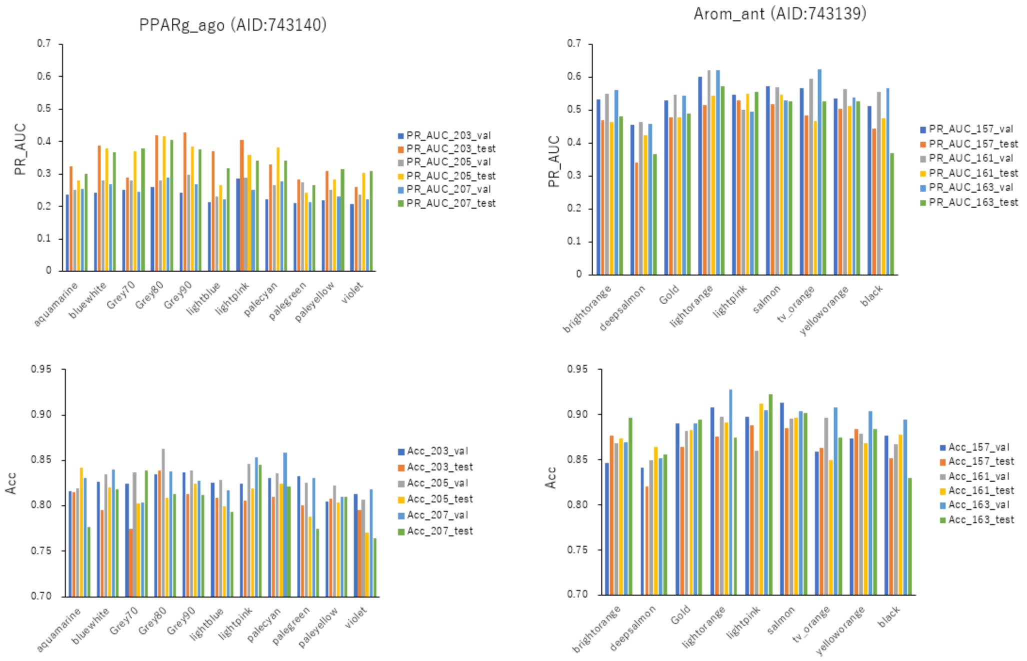

Figure S23. PR_AUC and Acc of PPARg_ago (AID:743140) and Arom_ant (AID:743139) by TensorFlow with background colors.

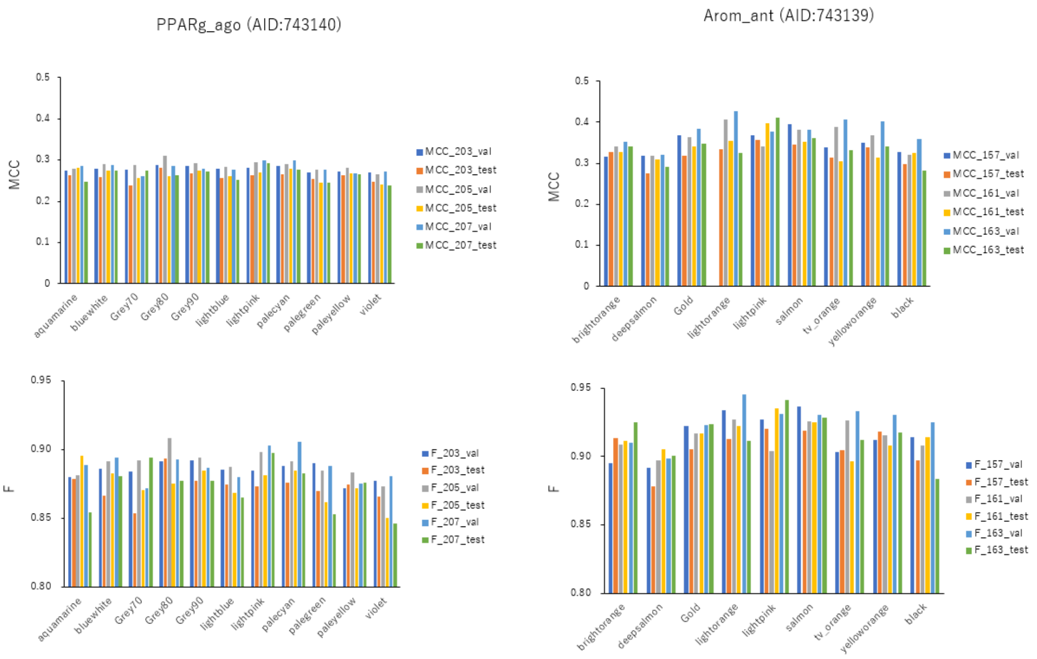

Figure S24. MCC and F of PPARg_ago (AID:743140) and Arom_ant (AID:743139) by TensorFlow with background colors.

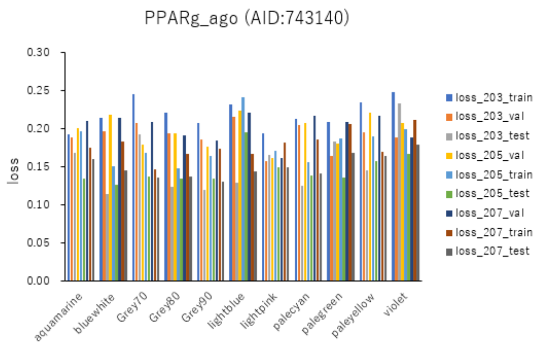

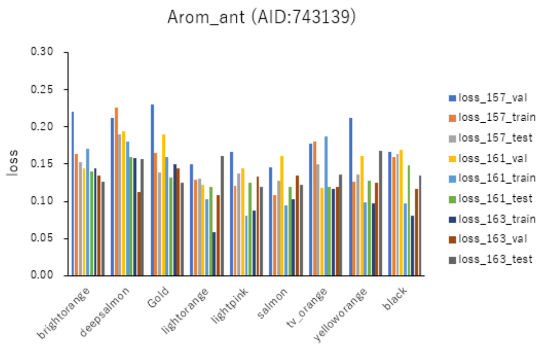

Figure S25. loss of PPARg_ago (AID:743140) and Arom_ant (AID:743139) by TensorFlow with background colors.
